# Designing Robust Superhydrophobic Materials for Inhibiting Nucleation of Clathrate Hydrates by Imitating Glass Sponges

**DOI:** 10.1021/acscentsci.2c01406

**Published:** 2023-02-10

**Authors:** Xinyu Yin, Yuanyang Yan, Xiangning Zhang, Bin Bao, Pihui Pi, Yahong Zhou, Xiufang Wen, Lei Jiang

**Affiliations:** † School of Chemical and Chemical Engineering, Guangdong Engineering Technology Research Center of Advanced Insulating Coating, South China University of Technology, Guangzhou 510640, People’s Republic of China; ‡ CAS Key Laboratory of Bio-inspired Materials and Interfacial Science, Technical Institute of Physics and Chemistry, Chinese Academy of Sciences, Beijing 100190, People’s Republic of China

## Abstract

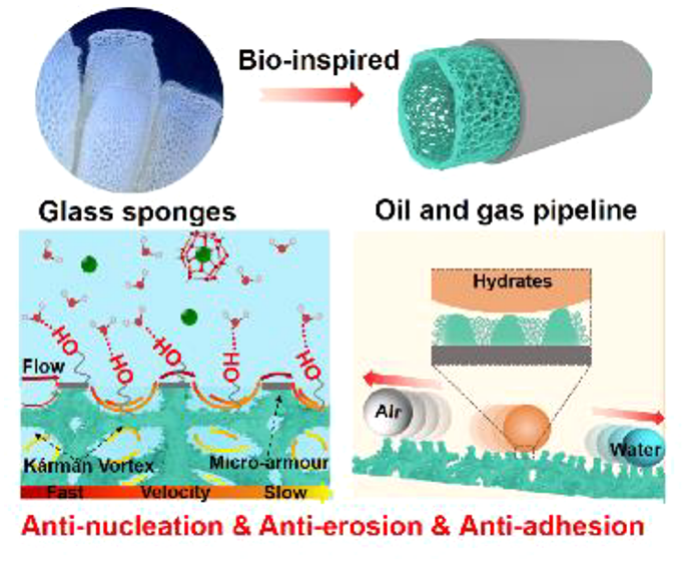

Superhydrophobic surfaces are suggested to deal with hydrate blockage because they can greatly reduce adhesion with the formed hydrates. However, they may promote the formation of fresh hydrate nuclei by inducing an orderly arrangement of water molecules, further aggravating hydrate blockage and meanwhile suffering from their fragile surfaces. Here, inspired by glass sponges, we report a robust anti-hydrate-nucleation superhydrophobic three-dimensional (3D) porous skeleton, perfectly resolving the conflict between inhibiting hydrate nucleation and superhydrophobicity. The high specific area of the 3D porous skeleton ensures an increase in terminal hydroxyl (inhibitory groups) content without damaging the superhydrophobicity, achieving the inhibition to fresh hydrates and antiadhesion to formed hydrates. Molecular dynamics simulation results indicate that terminal hydroxyls on a superhydrophobic surface can inhibit the formation of hydrate cages by disordering the arrangement of water molecules. And experimental data prove that the induction time of hydrate formation was prolonged by 84.4% and the hydrate adhesive force was reduced by 98.7%. Furthermore, this porous skeleton still maintains excellent inhibition and antiadhesion properties even after erosion for 4 h at 1500 rpm. Therefore, this research paves the way toward developing novel materials applied in the oil and gas industry, carbon capture and storage, etc.

## Abstract 2

Inspired by glass sponges, a robust porous superhydrophobic material with terminal hydroxyls was prepared to achieve inhibition to fresh hydrates and antiadhesion to formed hydrates.

## Introduction

Pipeline transportation has been the primary transportation mode of natural gas with reserves of (2–2.5) × 10^16^ m^3^, which could sustain our energy needs for almost 1000 years.^1,2^ However, natural gas molecules can be encapsulated in a cagelike hydrogen-bonded water cluster to form clathrate hydrate nuclei that grow and aggregate on the pipe wall, which results in hydrate blockage and huge losses in oil–gas transportation.^3^ To address this issue, most researchers have focused on developing chemical additives with inhibitory or antiaggregative functional groups (e.g., −OH etc.) to inhibit hydrate nucleation or prevent the formed hydrates from adhering to the pipe wall.^4−6^ Although the addition of inhibitors is an effective strategy, chemical additives pose issues regarding separation and recovery, environmental pollution, and high costs.^1,4^ It has been reported that hydrophilic surfaces with terminal hydroxyls could inhibit fresh hydrate nucleation,^7−9^ but there is still a risk of clogging the pipeline because the hydrophilic surfaces are able to increase the adhesion with the formed hydrates during transport.^10,11^ Recently, superhydrophobic materials have been proposed to mitigate hydrate plugging, since they could reduce the adhesion force between the formed hydrates and the pipe wall surfaces, preventing the aggregation and deposition of hydrate particles on the pipe walls.^12−16^ However, a superhydrophobic surface may promote the formation of fresh hydrate nuclei along the pipe walls by inducing an orderly arrangement of water molecules.^17−26^ Evidently, the paradox between antinucleation and antiadhesion of superhydrophobic surfaces seems to be irreconcilable. Moreover, the fragility of the superhydrophobic surfaces owing to the micro-/nanostructures limits their practical application.^27−30^ Therefore, developing robust superhydrophobic surfaces that can inhibit fresh hydrate nucleation while preventing formed hydrates form adhering to the pipe walls will be an effective strategy for managing hydrate blocking in pipelines.

The immobilization of abundant terminal hydroxyls on a superhydrophobic surface can improve its inhibition performance, but at the expense of superhydrophobicity and antiadhesion. A balance of the terminal hydroxyl content and the superhydrophobicity is the key to solve the problem. Glass sponges (*Euplectella aspergillum*) have three-dimensional (3D) porous skeletons with a high specific surface area which can increase the chances of predation.^31^ Besides, the skeletal motifs of glass sponges could reduce the overall hydrodynamic stress and flow speed,^32^ which considerably increase the durability of 3D superhydrophobic materials. Inspired by this phenomenon, we hold that preparing a glass-sponge-like superhydrophobic 3D porous skeleton is an effective strategy to address the aforementioned dilemma, for these skeletons could provide an ultrahigh specific surface area for the introduction of terminal hydroxyl groups to inhibit the nucleation of fresh hydrates and meanwhile maintain superhydrophobicity to reduce the adhesion of formed hydrates to the surface and improve the antierosion property of the materials.

**Figure 1:**
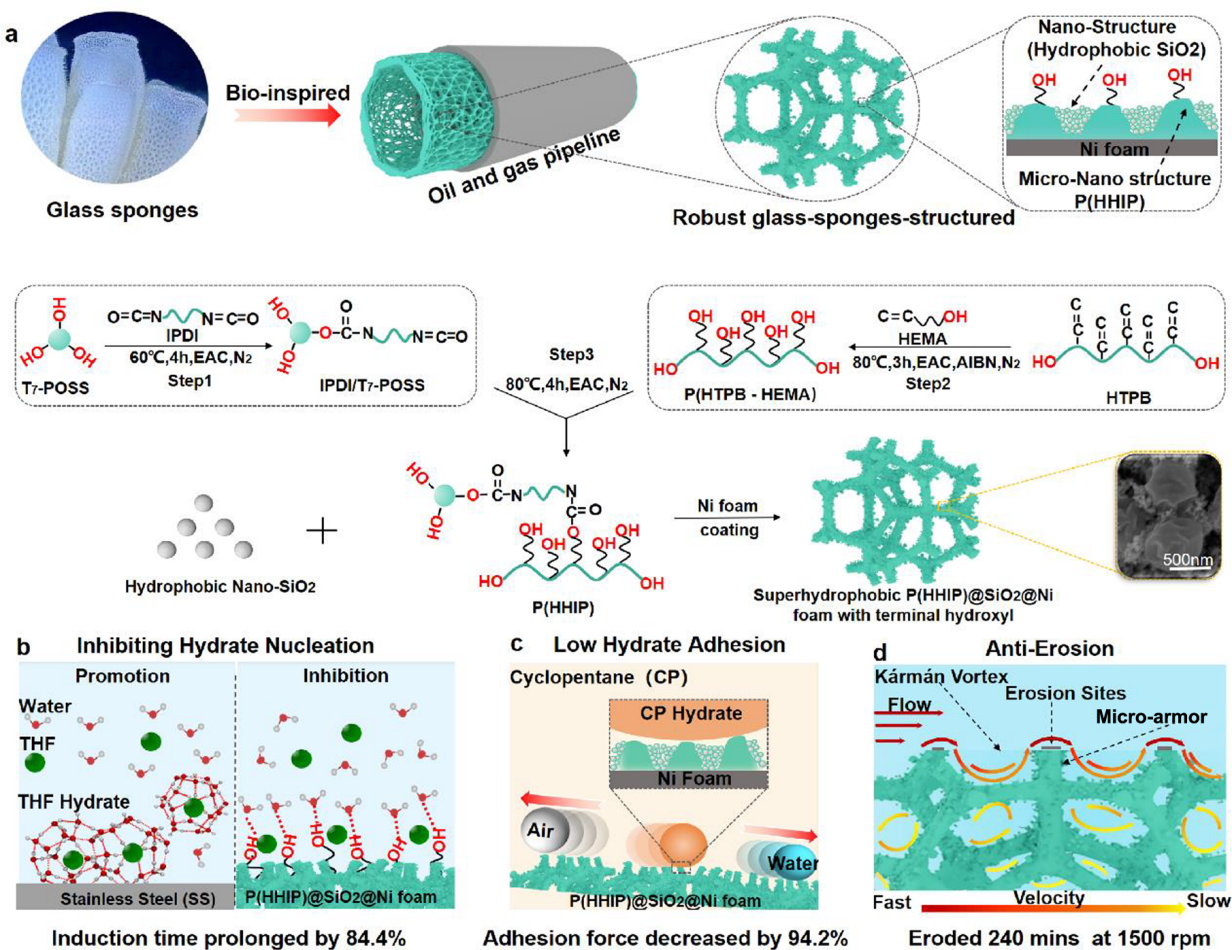
Glass-sponge-inspired superhydrophobic P(HHIP)@SiO_2_@Ni foam to inhibit hydrate nucleation and prevent hydrate adhesion and provide antierosion properties. (a) Schematic illustration showing the inspiration and strategy for preparing robust hydrate antinucleation superhydrophobic P(HHIP)@SiO_2_@Ni foam. Terminal hydroxyls are introduced onto the glass-sponge-like Ni foam with a high specific surface area. The inset images of positions marked by the gray dotted line delineate the 3D skeleton structures and terminal hydroxyls on its micro-/nanostructures, which are built by hydrophobic nano-SiO_2_ and P(HHIP) with inset images showing positions marked by the yellow dotted line. (b) Inhibition mechanism of the 3D P(HHIP)@SiO_2_@Ni foam. Enlarged hydroxyls form hydrogen bonds with water molecules and disorder the water arrangement. (c) The hydrate antiadhesion mechanism that minimizes the contact between hydrates. Inset: micro-/nanostructure on the Ni foam surface. (d) Antierosion mechanism that forms a Kármán Vortex to reduce the flow speed. The outer cavity of the skeleton protects the inner cavity from direct erosion.

Here, we successfully constructed a robust superhydrophobic 3D porous skeleton with inhibiting hydrate nucleating properties. As shown in Figure 1a, we synthesized the hydrophobic multihydroxyl polymer P(HHIP) that contains trisilanol phenyl POSS (T_7_-POSS) and hydroxyl-terminated side chains, which could disrupt the formation of hydrate cage formation to inhibit the formation of hydrates.^9,20^ Then a robust superhydrophobic 3D Ni foam with terminal hydroxyls (denoted P(HHIP)@SiO_2_@Ni foam) was prepared by coating Ni foam with a mixture of P(HHIP) and hydrophobic nano-SiO_2_. A molecular dynamics simulation predicted that introducing hydroxyl groups onto a superhydrophobic surface tended to inhibit the hydrate formation phenomenon. Our experimental data proved that the P(HHIP)@SiO_2_@Ni foam delayed the hydrate nucleation by disordering the uniform arrangement of water molecules and reduced the induction time of hydrate formation by 84.4%. Owing to the superhydrophobicity and superlipophilicity of P(HHIP)@SiO_2_@Ni foam, the adhesion force between hydrates and its surface was reduced by 98.7%. Moreover, the robust 3D porous skeleton could reduce the flow rate by forming a Kármán vortex, acting as “armor” to protect its internal body from erosion, which ensures its excellent inhibition and antiadhesion properties even after erosion for 4 h at a stirring rate of 1500 rpm. This research provides a new perspective on preparing robust materials for inhibiting hydrate nucleation and preventing the aggregation of hydrate particles on the walls of pipes for oil–gas transportation or grafting other functional groups to accelerate solidified natural gas and has potential in carbon capture and storage.

## Results and Discussion

### Thermodynamic Analysis and Molecular Dynamics Simulation Prediction

In industry, ethylene glycol is used to inhibit hydrate nucleation because its numerous hydroxyl groups reduce the nucleation temperature by forming hydrogen bonds with water molecules. According to the Hu–Lee–Sum (HLS) correlation^33^ (eq 1) and the relationship between hydrate inhibition temperature and water activity of the inhibitor aqueous solution^34^ (eq 2), hydrate nucleation can be inhibited by reducing the water activity, which can be further regulated by increasing the inhibitor concentration. Theoretically, increasing the concentration of hydroxyl groups on the pipeline surface can enhance the ability to inhibit hydrate nucleation at the interface.
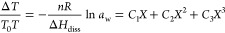
1
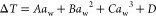
2Here, Δ*H*_diss_ denotes the hydrate dissociation enthalpy, *n* indicates the hydration number, *a*_w_ represents the water activity, *T*_0_ and *T* denote the hydrate dissociation temperatures for the fresh water and aqueous solution at a given pressure, respectively, Δ*T* (=*T*_0_ – *T*) represents the suppression temperature, *C*_1_, *C*_2_, and *C*_3_ denote the fitted coefficients, and *A*, *B*, *C*, and *D* denote constants that can be experimentally determined.

**Figure 2:**
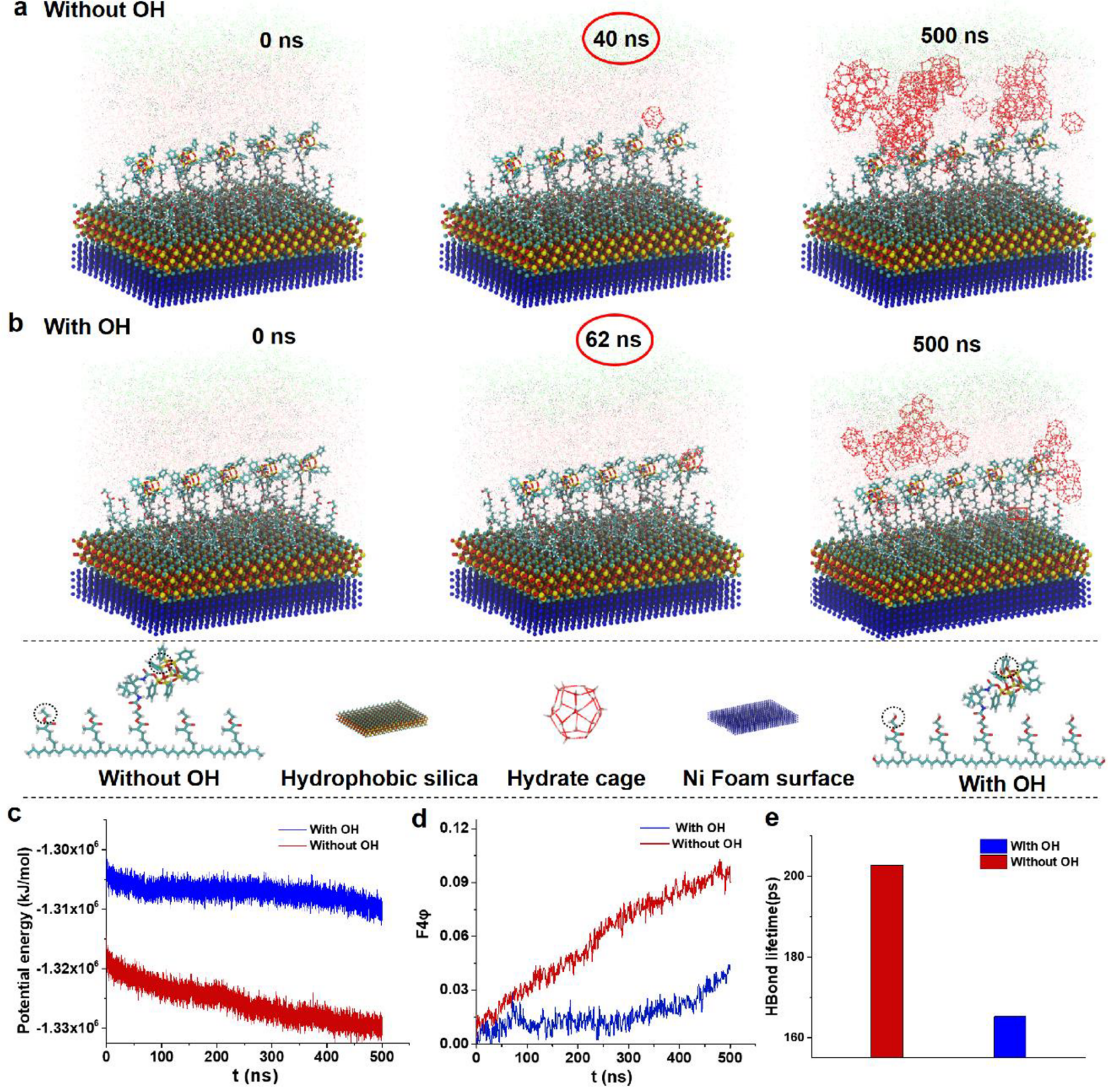
Molecular dynamics simulations predicting the effects of terminal hydroxyl groups on hydrate formation at 250 K and 500 bar. (a, b) Numbers of hydrate cages (red) formed during the hydrate nucleation process. (c) Comparison of variations in total potential energy with respect to time for multiple models representing the effect of hydroxyl groups on hydrate formation. (d) Comparison of the four-body order parameter F_4φ_ highlighting the effect of hydroxyl groups on hydrate formation. Degree of hydrate formation was quantified by *F*_4φ_. A greater amount of water was converted to hydrate cages for a large *F*_4φ_. (e) Influence of hydroxyl groups on the stability of hydrogen bonds during hydrate nucleation quantified by calculating the hydrogen-bonding lifetime between water and water.

Based on the stated analysis, we hypothesize that, instead of promoting nucleation, the superhydrophobic surface will inhibit nucleation upon the introduction of an adequate amount of hydroxyl groups with maintained superhydrophobicity. To validate the feasibility of this assumption, we conducted molecular dynamics simulations of hydrate formation on the superhydrophobic P(HHIP)@SiO_2_@Ni surface (the model with OH). In addition, we simulated a model without −OH in which the terminal hydroxyl was replaced by H atoms to understand the influence of the terminal hydroxyl segment on hydrate nucleation. The snapshots of the hydrate nucleation process are presented in Figure 2a,b. For the hydrate cage in the model without −OH appearing at 40 ns (Figure 2a), the number of hydrate cages exceeds 20 at 150 ns and thereafter increases almost exponentially (Figure S1a, red line). In contrast, in the model with −OH, the hydrate cage appeared at 62 ns (Figure 2b), and after 450 ns, the number of hydrate cages does not exceed 20 (Figure S1a, blue line). Thus, the terminal hydroxyl of P(HHIP)@SiO_2_@Ni surface evidently inhibits hydrate nucleation.

The total potential energy, four-body order parameter (*F*_4φ_) and hydrogen bond lifetime were calculated to reveal the hydrate nucleation inhibition mechanism of the terminal hydroxyls. As displayed in Figure 2c, the reduction in the total potential energy of the model with −OH (Figure 2c, dark blue line) gradually decreases in comparison to that of the model without −OH (Figure 2c, dark red line). This result implies that the hydroxyl delays the stabilizing tendency of the system, which potentially hinders the hydrate formation. Moreover, this can be attributed to a strong interaction (Figure S1b, dark blue line) resulting in the gradual reduction of the hydrate nucleation energy barrier. As a control group, the strong hydrophobicity of the model without −OH induces the orderly arrangement of water molecules (Figure S1b, dark red line), diminishes the energy barrier for hydrate nucleation, and promotes hydrate nucleation.^20,35,36^ The average *F*_4φ_ values for hydrate, liquid water, and ice are 0.7, −0.04, and −0.4, respectively.^37^ In particular, an *F*_4φ_ value greater than −0.04 indicates that more water molecules form hydrate cages. During the entire simulation process, the *F*_4φ_ value of the model with −OH (Figure 2d, dark blue line) gradually increases, whereas that of the model without −OH (Figure 2d, dark red line) increases drastically. More importantly, the *F*_4φ_ value of the model with −OH is overall less than that of the model without −OH, which is consistent with the evolution of the hydrate cages (Figure S1a). These results demonstrate that the terminal hydroxyl groups on the P(HHIP)@SiO_2_@Ni surface disorder the organization of the water molecules and prevent the formation of the hydrate cage at the interface.^38^ Furthermore, more hydrogen bonds form between the water molecules and the surface of the model with −OH (Figure S1c), and fewer hydrogen bonds are formed between the water molecules compared to that of the model without −OH (Figure S1d). These results manifest that the hydroxyl groups on the P(HHIP)@SiO_2_@Ni surface bonded with water molecules via hydrogen bonds (Figure S1e), and they affect the hydrogen bond formation between water molecules. In addition, the lifetime of the hydrogen bonds are calculated to quantify the influence of hydroxyl groups. The hydrogen bond stability is much stronger for a prolonged lifetime of the hydrogen bonds, which is conducive for constructing stable hydrate cages. As portrayed in Figure 2e, the hydrogen bonds in the model with −OH exhibited a shorter lifetime compared to that of the model without −OH, which further establishes the pivotal role of terminal hydroxyl groups.

Conclusively, the terminal hydroxyl groups on P(HHIP)@SiO_2_@Ni surface could bond to water and disorder the organization of water molecules to defer the formation of hydrate clathrate at the interface. In addition, it indicates that inhibiting hydrate nucleation and superhydrophobicity can be simultaneously achieved on a superhydrophobic surface.

### Characterization of P(HHIP)@SiO_2_@Ni Foam

**Figure 3:**
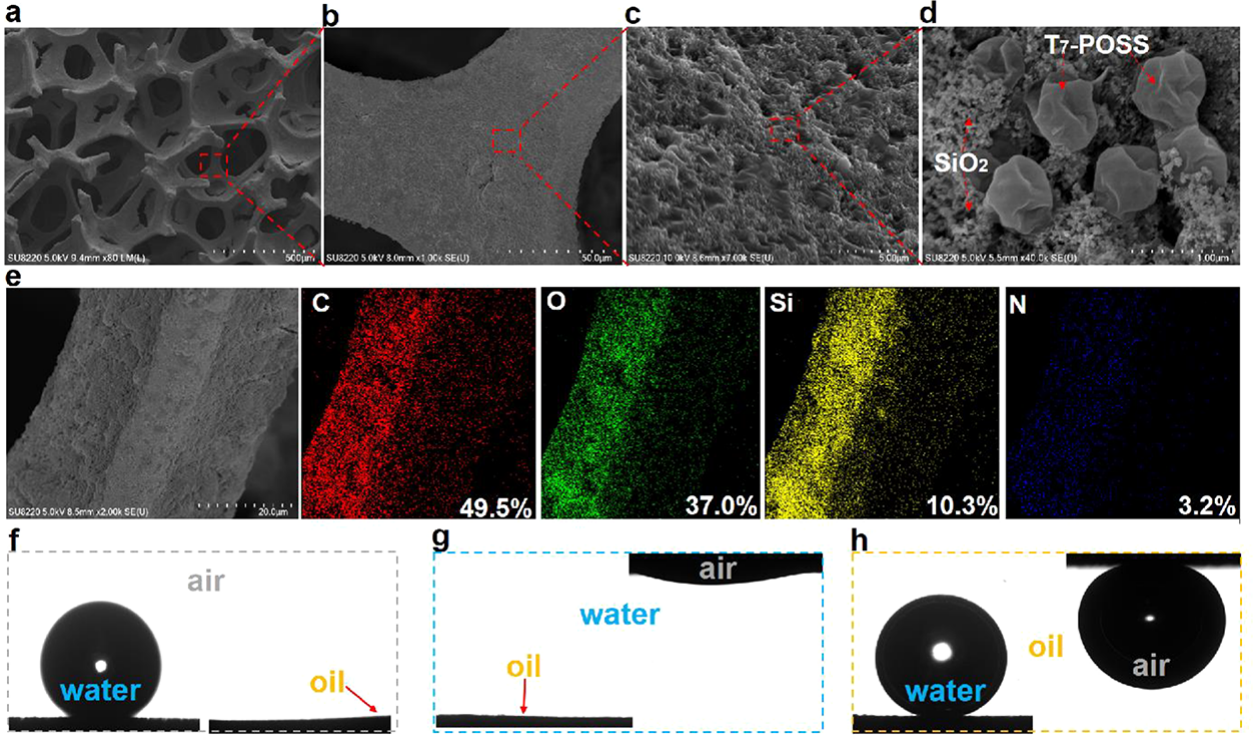
Structural and wettability characteristics of the P(HHIP)@SiO_2_@Ni foam. (a–d) Scanning electron micrographs displaying the topography of the P(HHIP)@SiO_2_@Ni foam: (a, b) low-magnification SEM images exhibiting the surface morphology of the microscale skeleton; (c, d) high-magnification SEM images displaying the micro-/nanostructure topography constructed by nano-SiO_2_ and T_7_-POSS. (e) EDS mapping of P(HHIP)@SiO_2_@Ni foam illustrating the dispersion of elements on its surface. (f–h) Images indicating the wettability of P(HHIP)@SiO_2_@Ni foam in various environments: (f) superhydrophobicity and superlipophilicity in air; (g) superlipophilicity and superaerophilicity under water; (h) superhydrophobicity and superaerophobicity in oil.

Inspired by the extremely strong skeleton of glass sponges and the high specific surface area generated by the mineralized nanosilica and organic interlayers,^39^ we selected 3D porous robust Ni foam as the skeleton. As depicted in Figure 1a, the synthesized multihydroxyl polymer P(HHIP) serves as the “organic interlayers” containing T_7_-POSS and hydroxyl-terminated side chains, which is proved by the Fourier-transform infrared (FT-IR) spectra (Figure S2a). In addition, the micro-/nanostructures were constructed by dipping Ni foam into a mixture of P(HHIP) and hydrophobic nanosilica, which enabled the dynamic modulation of the hydrophobicity and hydroxyl content. As displayed in Figure 3a,b, the 3D porous skeleton structures of P(HHIP)@SiO_2_@Ni foam were characterized by scanning electron microscopy (SEM). The “armor-like”^40^ micro-/nanostructures (Figure 3c,d) with high specific surface area are favorably built on its surface by nanosilica and P(HHIP) (Figure S2b,c). Moreover, the mapping images obtained from energy-dispersive X-ray spectroscopy (EDS) (Figure 3e) also indicate the uniform P(HHIP)@SiO_2_ coating on the Ni foam surface.

The static (Figure 3f) and dynamic (Figure S2d,e) contact angle (CA) tests signified the excellent superhydrophobicity of the P(HHIP)@SiO_2_@Ni foam, which even could bounce a water jet (Figure S3a). Besides, oil droplets (5 μL dichloroethane) rapidly wet the surface of the P(HHIP)@SiO_2_@Ni foam within 0.05 s (Figure S2f) at a CA of 0° (Figure 3f), demonstrating its outstanding superlipophilicity in the air. Moreover, it was wetted by oil droplets even in water at a CA of almost 0° (Figure 3g), and the oil droplets expelled the air in their body cavities, forming large air bags due to the superlipophilicity (Figure S3b). This manifests the superlipophilicity of the P(HHIP)@SiO_2_@Ni foam under water. When the P(HHIP)@SiO_2_@Ni foam was immersed in oil, the CA of water on its surface was 155 ± 2° (Figure 3g). The water droplets could bounce off with a V-shaped water column and could not penetrate the material (Figure S3d), implying the superhydrophobicity of the P(HHIP)@SiO_2_@Ni foam in oil.

### 3D Porous Skeleton Enhancing Inhibition Performance by Increasing Hydroxyl Content

**Figure 4:**
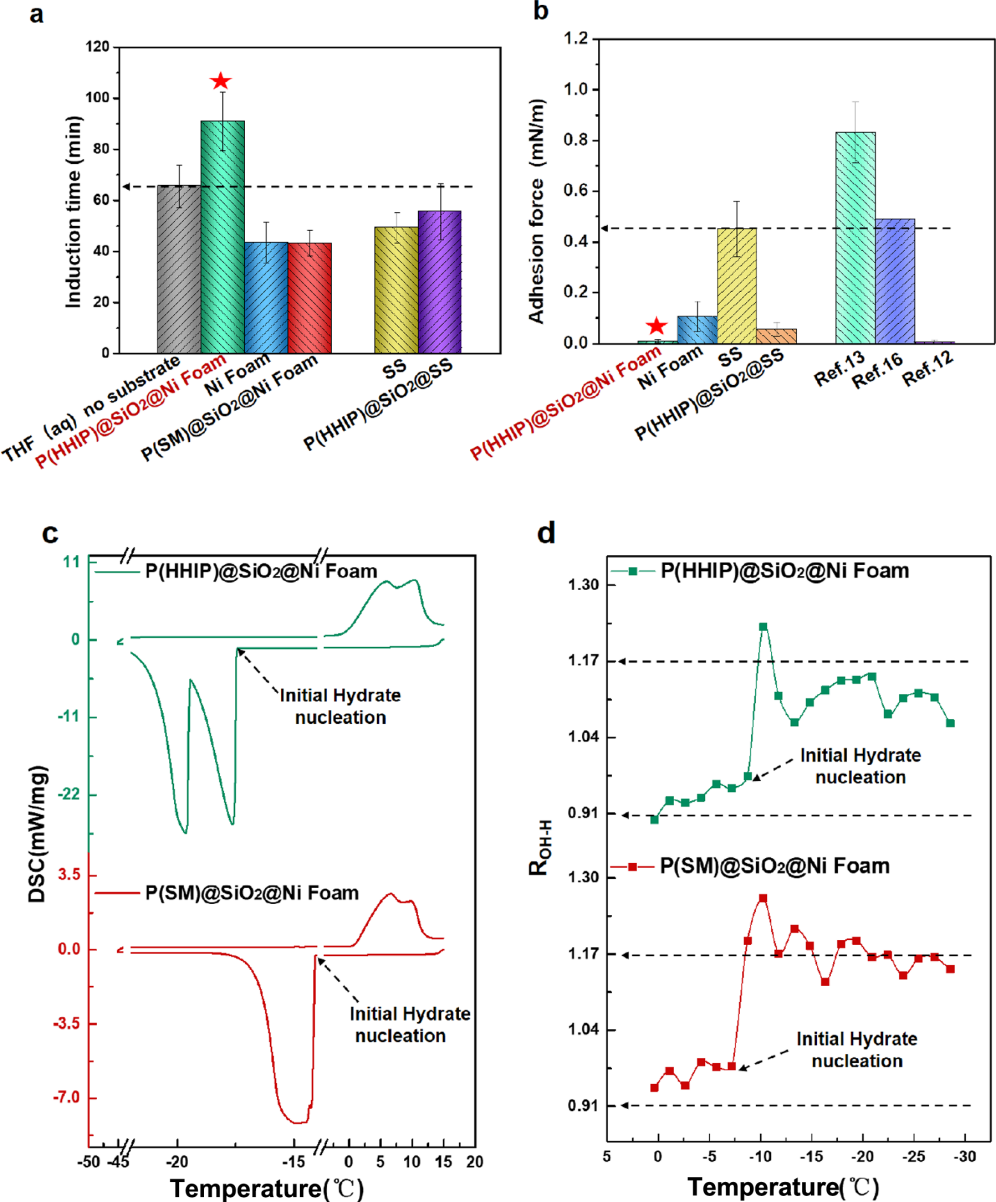
Evaluation of the inhibition nucleation and antiadhesion performance of the P(HHIP)@SiO_2_@Ni foam and analysis of inhibition mechanism. (a) Comparison of mean induction time of samples highlighting the vitality of the 3D porous skeleton and terminal hydroxyls for the inhibition of hydrate nucleation (Δ_subcooling_ = 4.4 °C; stirring rate 300 rpm). (b) Comparison of mean hydrate adhesion force between CP hydrate particles and sample surfaces representing the advantage of the 3D porous skeleton with micro-/nanostructures (Δ_subcooling_ = 4.5 °C). (c) Comparison of the exothermic curves of samples showing the effects of hydroxyl groups on hydrate nucleation temperature. (d) Comparison of the *R*_OH_ curves of different samples showing the effects of hydroxyl groups on the order of water molecules. Error bars in all graphs represent the standard deviation of the mean.

The influences of terminal hydroxyls and the 3D skeleton structure on inhibiting hydrate nucleation was characterized by the induction time of hydrate nucleation (Figures S4a and S5). As depicted in Figure 4a, compared to the induction time of uncoated stainless steel (denoted as SS), Ni foam promotes hydrate nucleation by 11.8%; meanwhile the superhydrophobic P(HHIP)@SiO_2_@Ni foam prolongs the induction time by 84.4%. Notably, P(stearyl methacrylate-*co*-MAPOSS)@SiO_2_@Ni foam, which has the same types of functional groups as P(HHIP)@SiO_2_@Ni foam with the exception of terminal hydroxyls, reduces the induction time by 12.2%. These results confirm that the terminal hydroxyl groups on a superhydrophobic surface inhibit hydrate nucleation, which is in accordance with the current simulation results.

In addition, the uncoated 3D Ni foam facilitates hydrate nucleation compared to uncoated SS ; however, after being modification by P(HHIP)@SiO_2_ coating , the high surface area of the 3D porous skeleton allows more terminal hydroxyl groups on its superhydrophobic surface, resulting in a 38.8% improvement in hydrate inhibition of superhydrophobic P(HHIP)@SiO_2_@Ni foam over P(HHIP)@SiO_2_@SS. Thus, these results confirm the vitality of 3D porous skeletons for balancing mutual benefits between antinucleation and superhydrophobicity, which is consistent with our simulations and ultimately verifies our hypothesis. Besides, we quantitatively accessed the effect of organic matter on hydrate inhibition performance and the suppression performance of ambient stored samples; the results show that neither has a significant effect on the induction time of P(HHIP)@SiO_2_@Ni foam (Figure S5b,c). In addition, a visual experiment further confirms that the P(HHIP)@SiO_2_@Ni foam inhibits the formation of hydrates (Figure S6).

To unveil the hydrate-nucleation inhibition mechanism of the P(HHIP)@SiO_2_@Ni foam, we conducted *in situ* differential scanning calorimetry (DSC). As indicated in Figure 4c, the hydrate nucleation temperature of the P(HHIP)@SiO_2_@Ni foam (−17.4 °C) is less than that of P(SM)@SiO_2_@Ni foam (−14.3 °C). This was attributed to the strong hydrogen bonding interaction between terminal hydroxyls and water molecules as predicted by molecular dynamics simulations that decreased the nucleation temperature by hydrogen bonding with water molecules. And the exothermic curve of the P(HHIP)@SiO_2_@Ni foam (Figure 4c, dark green line) exhibiting two narrow peaks may indicate that ice cluster formation and hydrate nuclei nucleation are completed in stages, while broad exothermic peaks (Figure 4c, dark red line) exist for P(SM)@SiO_2_@Ni foam. These results further proved the terminal hydroxyls can delay hydrate nucleation.

Furthermore, we employed *in situ* Raman spectra to record the variations in peak intensity ratio of various samples (*R*_OH_ = *I*_3200_/*I*_3400_) during hydrate formation (Figure S7a(i),b(i)).^22,41,42^ In principle, more water molecules are organized in the case of larger *R*_OH_ values, which reduces the energy barrier and accelerates hydrate nucleation.^17,19−21,23−26,43^ In particular, *R*_OH-–W_ and *R*_OH-H_ represent the order of water molecules in water (Figure S7a(ii),b(ii)) and THF solutions (Figure S7a(iii),b(iii)), respectively.

As depicted in Figure 4d, as the temperature decreases further, the formation of hydrates is inevitable under a high-undercooling condition, but the *R*_OH_ curves of the two samples are significantly different, as follows: the *R*_OH-H_ value of the P(SM)@SiO_2_@Ni foam gradually increases until a large amount of hydrate cages form (for the first peak, *R*_OH–H_ = 1.27 while the *R*_OH-H_ value of the P(HHIP)@SiO_2_@Ni foam is 1.23). As the subcooling continues to increase, *R*_OH-H_ of the P(SM)@SiO_2_@Ni foam exhibits fluctuations at ∼1.17 until the hydrates completely nucleate, but the *R*_OH-H_ value of the P(HHIP)@SiO_2_@Ni foam increased slowly and is less than 1.17. For the P(SM)@SiO_2_@Ni foam, due to its strong hydrophobic hydration, induces the orderly arrangement of water molecules to form fresh hydrates, increasing *R*_OH-H_ while the heat released by formation of fresh hydrates decreased *R*_OH-H_. But for the P(HHIP)@SiO_2_@Ni foam, owing to the fact the terminal hydroxyl groups could bond to water molecules and disorder the arrangement of water molecules, its *R*_OH–H_ is smaller than that of P(SM)@SiO_2_@Ni foam during the nucleation process, and its initial nucleation temperature is also lower than that of the P(SM)@SiO_2_@Ni foam, which coincides with their DSC curves. Besides, the Δ*R*_OH_ values (Δ*R*_OH_ = *R*_OH-H_ – R_OH-W_; please refer to Figure S7 for details of adhesion calculations) of the P(HHIP)@SiO_2_@Ni foam and P(SM)@SiO_2_@Ni foam are 0.18 and 0.08, respectively, which is consistent with the results reported by Li et al.^41^—larger values of Δ*R* prolong the hydrate induction time.

Based on the simulation and experimental results, the possible inhibition mechanism is exemplified in Figure 1d. Owing to high subcooling, water molecules are driven to organize hydrate nuclei, and the uncoated SS (or Ni foam) promotes hydrate heterogeneous nucleation by providing nucleation sites. In contrast, P(HHIP)@SiO_2_@SS and P(HHIP)@SiO_2_@Ni foam exhibit an inhibiting effect on hydrate nucleation, which can be attributed to the terminal hydroxyl groups forming hydrogen bonds with water molecules, disorienting the arrangement of water molecules and delaying the complete formation of the hydrate cage.^44,45^ Compared to P(HHIP)@SiO_2_@SS, the P(HHIP)@SiO_2_@Ni foam with a high specific surface area provides more terminal hydroxyl groups, and consequently, the strong interaction between the terminal hydroxyls and water molecules decreases the nucleation temperature and disrupts the formation of a hydrate cage.

### 3D Porous Superhydrophobic and Superlipophilic Skeleton Reducing Hydrate Adhesion

To quantify the antiadhesion performance of the P(HHIP)@SiO_2_@Ni foam, we measured the adhesion force between the cyclopentane (CP) hydrate and the surfaces of various samples (Figures S4b and S8, please refer to Supplementary Section 3 for details of adhesion calculations). As observed from Figure 4b, the mean values of the adhesion force of CP hydrates on the uncoated SS, Ni foam, P(HHIP)@SiO_2_@SS, and P(HHIP)@SiO_2_@Ni foam were 0.449, 0.104, 0.053, and 0.006 mN/m, respectively. Compared to uncoated SS, the adhesion force of the P(HHIP)@SiO_2_@Ni foam was reduced by 98.7%. Evidently, the P(HHIP)@SiO_2_@Ni foam remarkably reduces the adhesion strength.

The eminent antiadhesion performance of the P(HHIP)@SiO_2_@Ni foam could be summarized as being due to three major reasons. First, the micro-/nanostructures constructed by T_7_-POSS and hydrophobic SiO_2_ reduce the contact area between the hydrate particles and the sample surface (Figure 1c and Figure 3d). This also explains that the adhesion force of uncoated Ni foam or SS is much larger than that of P(HHIP)@SiO_2_@Ni foam or P(HHIP)@SiO_2_@SS. Second, the protruding ridges of the microskeleton (Figure 3a) contacted the hydrate particles following a point-to-point basis (Figure S8c,d), whereas the hydrate particles and P(HHIP)@SiO_2_@SS are in point-to-surface contact (Figure S8a,b), owing to which the resulting adhesion force on P(HHIP)@SiO_2_@SS is 8.8 times larger than that on the P(HHIP)@SiO_2_@Ni foam. Evidently, compared to the reported planar materials (Table S1), the superiority of 3D porous materials in preventing hydrate adhesion on a pipeline surfaces is much more notable. Third, the superlipophilicity of the P(HHIP)@SiO_2_@Ni foam is responsible for its lower adhesion force in comparison to alternative superhydrophobic materials reported in the literature.^12,13,16^ This is because the P(HHIP)@SiO_2_@Ni foam surface could be rapidly wetted using cyclopentane within 0.05 s and form a cyclopentane barrier film to isolate the hydrate particles from its surface, which is consistent with Das’ findings.^46^

### 3D Porous Skeleton Enhances Erosion Resistance

**Figure 5:**
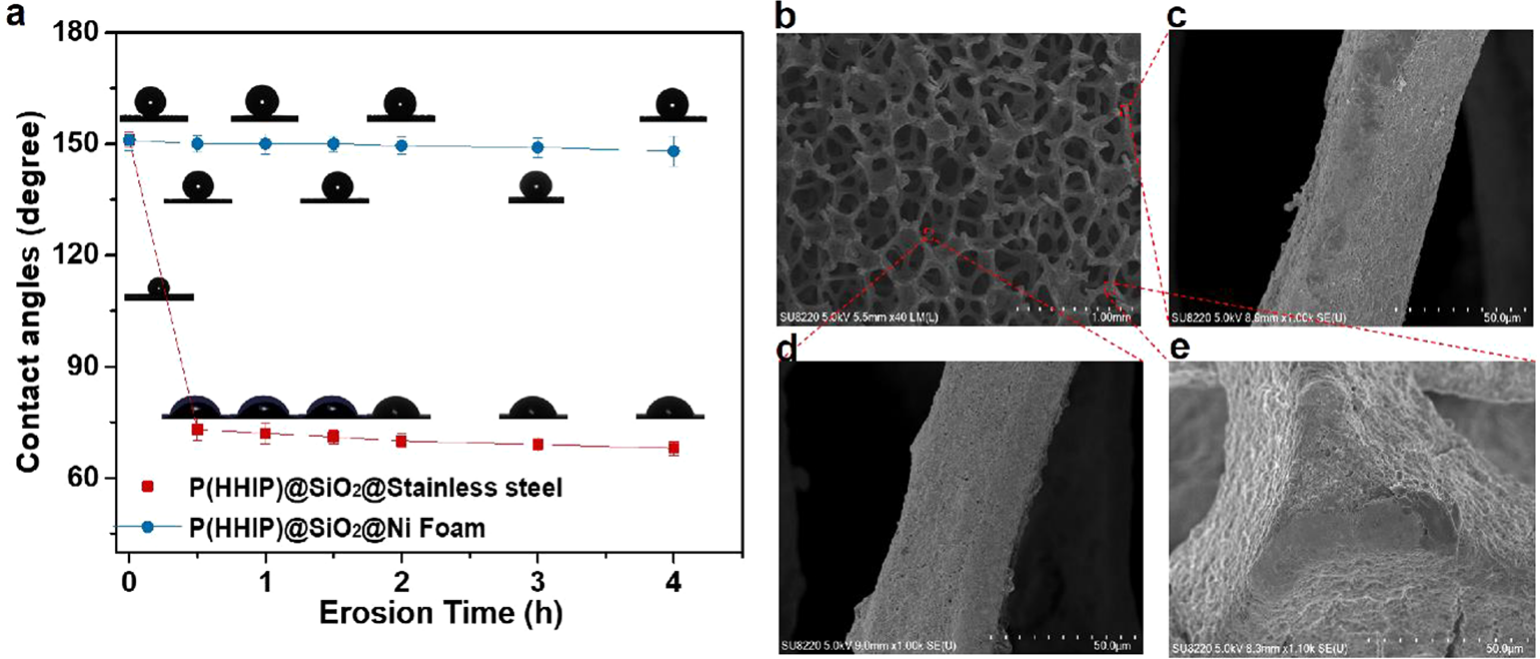
Evaluation of erosion resistance of P(HHIP)@SiO_2_@Ni foam. (a) Characterization of antierosion by measuring a series of contact angles of the P(HHIP)@SiO_2_@Ni foam and P(HHIP)@SiO_2_@SS with versus erosion times. (b–e) Scanning electron micrographs displaying the skeleton of P(HHIP)@SiO_2_@Ni foam after erosion for 4 h, and its local enlarged micrographs: (c) exterior ridges of skeleton, (d) inner ridges of skeleton, and (e) protruding skeleton of P(HHIP)@SiO_2_@Ni foam. Error bars in all graphs represent the standard deviation of the mean.

To verify erosion resistance, the P(HHIP)@SiO_2_@Ni foam and the P(HHIP)@SiO_2_@SS (as control group) were eroded with a sand-containing water at a stirring speed of 1500 rpm (Figure S9 and Supplementary Video 1). After continuous erosion for 4 h, the P(HHIP)@SiO_2_@Ni foam retained its superhydrophobicity (Figure 5a, blue line; Figure S10a,c), whereas the coating on the surface of P(HHIP)@SiO_2_@SS was almost worn out and lost its superhydrophobicity within 15 min (Figure 5a, red line; Figure S9b). To prove the erosion resistance of the P(HHIP)@SiO_2_@Ni foam, a scratch test (at least 80 times) and a friction test (at least 60 times) were carried out (for details refer to Supplementary Section 4 of the Supporting Information). After the above tests (Supplementary Videos 2 and 3), the abraded surface still allowed water drops to roll off without residues. The contact angle tests showed no significant change in superhydrophobicity of the P(HHIP)@SiO_2_@Ni foam before and after abrasion tests (Figure S10b). Besides, the P(HHIP)@SiO_2_@Ni foam surfaces demonstrated favorable chemical stability when it was exposed to solutions with pH values from 1 to 10 (Figure S11). These results validate the excellent antierosion properties of the P(HHIP)@SiO_2_@Ni foam.

The reasons for the high erosion resistance of P(HHIP)@SiO_2_@Ni foam can be stated as follows: first, similarly to the skeletal motifs of glass sponges,^32^ the ridges and body cavity of the P(HHIP)@SiO_2_@Ni foam (Figure 1c) reduce the hydrodynamic stress and consume the erosion on the material surface by forming a Kármán vortex to weaken the flow velocity.^31^ Similarly, with the microframes acting as “armor”,^40^ they could prevent the erosion of the interior body cavities, while only the ridges and exterior body cavities of P(HHIP)@SiO_2_@Ni foam were slightly damaged and the micro-/nanostructures of inner body cavities exhibited no substantial deformations (Figure 5c–e). Thus, the induction time of P(HHIP)@SiO_2_@Ni foam is 72.9% longer than that of uncoated SS and retains its superhydrophobicity even after erosion for 4 h (Figure S5b,c).

## Conclusions

In summary, inspired by the strong 3D skeleton of glass sponges, we formulated new strategies in this study to fabricate a robust superhydrophobic P(HHIP)@SiO_2_@Ni foam that simultaneously inhibited fresh hydrate nucleation and reduced the adhesion between the formed hydrates and the material surface. A molecular dynamics simulation predicted that the formation of hydrates could be inhibited by introducing hydroxyl groups onto the superhydrophobic surface. Our experimental data proved that the superhydrophobic P(HHIP)@SiO_2_@Ni foam with terminal hydroxyls delayed the hydrate nucleation by disordering the uniform arrangement of water molecules and prolonged the induction time of hydrate formation by 84.4%. Due to the superhydrophobicity and superlipophilicity of P(HHIP)@SiO_2_@Ni foam, the adhesion force between hydrates and its surface was reduced by 98.7%. Moreover, the robust 3D porous skeleton could reduce the flow rate by forming a Kármán vortex, acting as “armor” to protect its internal body from erosion, which ensured its excellent inhibition performance and antiadhesion properties even after erosion for 4 h at a stirring rate of 1500 rpm. This can be attributed to the 3D skeleton structures that not only balance a mutually beneficial relationship between antinucleation and the superhydrophobic surface but also improve erosion resistance by forming a vortex. Thus, this study establishes a new strategy for the development of multifunctional materials, which will be advantageous in fields ranging from oil–gas storage and transportation to carbon capture and storage, reduction of carbon emissions, and beyond.

## Experimental Section

### Molecular Dynamics Simulations

As displayed in Figures 2a,b, the box dimensions were 5.9 nm × 8.1 nm × 8.5 nm. From the bottom to the top, the material was composed of Ni foam, hydrophobic silica, and P(HHIP). The remainder of the box was filled with THF solution, and a small amount of methane was added to the gas and liquid phases to promote the nucleation of the solution and reduce the consumption of the computing resources.^47^ To further understand the inhibiting mechanism, all of the hydroxyl groups in P(HHIP) were replaced with hydrogen, which served as a control group. Specifically, the liquid phase in the box contained 344 THF molecules, 6440 water molecules, and 300 methane molecules, and the gas phase contained 550 methane molecules. The mass fraction of the tetrahydrofuran solution was consistent with the experiment. All molecules were all-atom models except for the methyl groups on the silica surface. Subsequently, the OPLS-AA force field^48^ was used to calculate the molecular interactions among nickel, hydrophobic silica, P(HHIP), THF, water, and methane. In particular, the potential parameters for the nonbonded interactions of nickel and hydrophobic silica were obtained with reference to Heinz et al.^49^ and Ji et al.,^50^ respectively. Moreover, the geometry of P(HHIP) was submitted to PolyParGen to obtain its topology that is compatible with the OPLS-AA force field parameter set.^50^ In addition, we used TIP4P-ICE for water,^51^ OPLS-AA for methane,^52^ and modified model 7 from Girard et al.^53^ for THF. Thereafter, short energy minimization and NPT simulation were performed to relax the system and eliminate the unreasonable contact of the model system. Ultimately, the system was sampled for 500 ns in the NPT ensemble, using a Berendsen barostat and V-rescale thermostat to maintain the pressure at 500 bar and the temperature at 250 K by GROMACS (version 5.1.5), respectively.^54^ All simulations were performed under a *xy*-dimensional periodic boundary condition; however, the *z*-dimension was not periodic owing to the virtual walls filled with oxygen atoms at the top and bottom. Furthermore, the algorithm developed by Mahmoudinobar et al.^55^ was used to calculate the amount of hydrate nucleation in both systems during simulations.

### Materials

Trisilanol phenyl POSS (T_7_-POSS), 2-hydroxyethyl methacrylate (HEMA), isophone diisocyanate (IPDI), 2,2-azobis(2-methylpropionitrile) (AIBN), tetrahydrofuran (THF, 99.5 wt %) and *N*,*N*-dimethylformamide (DMF, 99.5 wt %) were procured from Aladdin Reagent Co. (Shanghai, China). Ethyl acetate (EA), acetone, and diethyl ether were sourced from Guangzhou Cong Yuan Chemical Co. Ltd., China. Hydroxyl-terminated polybutadiene propellant (HTPB) was supplied by Tian Yuan Chemical Research Institute New Material Incubator Co., Ltd., China. Hydrophobic silica (20–60 nm) was provided by BiSheng Ji Chemical Co. Ltd., China. The curing agent (N95) was procured from Covestro Reagent Co. (Shanghai, China). Except for the indicated reagents, all of the aforementioned reagents were analytical grade reagents.

### Preparation of P(HHIP)@SiO_2_@Ni Foam

The preparation process of P(HHIP) and P(HHIP)@SiO_2_@Ni foam is illustrated in Figure 1a, and the details are presented in the Supporting Information.

### Characterization

Tetrahydrofuran (THF) and cyclopentane (CP) are commonly used to evaluate the inhibition and antiadhesion properties of additives, respectively,^7,12,16,46,56^ because they can form the most common gas hydrate structure (structure II) under atmospheric conditions while other small hydrocarbons require high pressure due to their poor solubility in water. An experimental apparatus (Figure S4a) was utilized to measure the induction time of THF hydrate nucleation. The adhesion force between the sample surfaces and hydrate was tested using micromechanical force (Figure S4b). Additionally, the test details and calculations of the induction time and adhesion force are presented in the Supporting Information. The surface wettability of the samples was measured using a CA analyzer (Date Physics OCA20, Germany). Moreover, FTIR spectra were recorded on a Bruker VERTEX 70 spectrometer, and a SEM (Zeiss Merlin, Germany) was used to observe the surface morphology. The surface elements and composition were determined using an energy dispersive spectrometer (EDS, X-Max N20, Oxford). The ordering of water molecules on the sample surfaces was recorded with a Raman spectrometer (Renishaw) using a laser wavelength of 532 nm. In particular, a DSC 214 (NETZSCH) instrument was utilized to obtain the heat flow curve of hydrate formation and dissociation process. For in situ Raman spectroscopy, DSC, and the erosion test procedures, please refer to the Supporting Information.

## Supporting Information Available

The data that support the findings of this study are available in the Supporting Information of this article. This PDF file includes: Supplementary Text 1–4; Supplementary Figures 1–11; Supplementary Table S1 (the summary of antiadhesion performance of superhydrophobic materials); Supplementary Videos 1–3; References 1–14. The Supporting Information is available free of charge at https://pubs.acs.org/doi/10.1021/acscentsci.2c01406.

Design and characterization of P(HHIP)@SiO_2_@Ni foam, inhibiting hydrate nucleation test, antiadhesion test, mechanical and chemical stability tests (PDF)
Antierosion test (MP4)
Antiscratch test (MP4)
Antiabrasion test (MP4)
