# Supplementary material for: Designing Robust Superhydrophobic Materials for Inhibiting Nucleation of Clathrate Hydrates by Imitating Glass Sponges
Source: ACS Cent Sci. 2023 Feb 10;9(2):318–27. doi: 10.1021/acscentsci.2c01406 (PMC9951277; doi:10.1021/acscentsci.2c01406)
Supplement: Supplementary file 1 — oc2c01406_si_001.pdf [file oc2c01406_si_001.pdf]

Supporting Information

Designing robust superhydrophobic materials for inhibiting nucleation of clathrate hydrates by imitating glass-sponges

Xinyu Yin,[1†] Yuanyang Yan,[1†] Xiangning Zhang,[1] Bin Bao,[2] Pihui Pi,[1] Yahong Zhou,[2] Xiufang Wen[1*] and*

Lei Jiang

1 School of Chemical and Chemical Engineering, Guangdong Engineering Technology Research Center of Advanced Insulating Coating, South China University of Technology, Guangzhou 510640, PR China
2 CAS Key Laboratory of Bio-inspired Materials and Interfacial Science, Technical Institute of Physics and Chemistry, Chinese Academy of Sciences, Beijing 100190, PR China
†These authors contribute equally to this work.
*Correspondence:
zhouyh@mail.ipc.ac.cn (Y. Zhou), xfwen@scut.edu.cn (X. Wen).

This PDF file includes:

Supplementary Text (1-4):
Supplementary Section 1: Design and Characterization of P(HHIP)@SiO_2@Ni foam
Supplementary Section 2: Inhibiting hydrate nucleation test
Supplementary Section 3: Anti-hydrate-adhesion test
Supplementary Section 4: Mechanical and chemical stability tests
Figures S1 to S9
Table S1
Supplementary Videos (1-3)
Supplementary Video 1： Anti-erosion test.
Supplementary Video 2： Anti-scratch test.
Supplementary Video 3： Anti-abrasion test.
References1-14

Supplementary Section 1: Design and Characterization of P(HHIP)@ SiO_2@Ni foam

1.1 Synthesis progress of P(HHIP):

Firstly, a flask was charged with 2g of HTPB (0.035mol C=C) dissolved in ethyl acetate and the initiator 2,2-azobis(2-methylpropionitrile) (AIBN). Then, using a constant pressure separating funnel, HEMA (0.035 mol) dissolved in ethyl acetate was added dropwise, and the mixture was allowed to react for 3 hours at 80 °C. The reaction yielded was named HTPB-co-HEMA. Secondly, a flask was charged with 2 g of T_7-POSS (0.003 mol) dissolved in ethyl acetate. Then, using a constant pressure separating funnel, IPDI ethyl acetate solution (0.035 mol) was added dropwise, and the mixture was allowed to react for 3 hours at 60 °C. The reaction yielded was named IPDI/T_7-POSS. Lastly, the HTPB-co-HEMA was put to a flask, and the IPDI/T_7-POSS was added dropwise using a constant pressure separating funnel. The mixture was then allowed to react at 80 °C for 3 hours. The reaction yielded was named P(HHIP).

1.2 Preparation of P(HHIP)@ SiO_2@Ni foam:

Pretreatment of the substrate: Surface contaminants and oxides were removed by sonicating Ni foam (110ppi) with a size of 1cm×2cm×0.13cm in acetone, ethanol, 0.1 M hydrochloric acid, and deionized water for 10 min each.

Preparation of P(HHIP)@SiO_2@Ni foam: The mixture of 3ml P(HHIP), 1ml ethyl acetate, and 1w% hydrophobic silica were sonicated for 30 mins. Then dip the cleaned Ni foam into the mixture and dry at 85 °C for 2 h, obtaining P (HHIP)@SiO_2@Ni foam.

1.3 Characterization of P(HHIP)@SiO_2@Ni foam

The dark red line in Figure S2a represented the FTIR spectra of HTPB, with a wide band at 3364 cm^{-1}, caused by the OH stretching vibration of the terminal hydroxyl group of HTPB; C=C double bond characteristic peaks were at 1642cm^{-1}; and 1,4 and 1.2 cis-trans olefin double bond characteristic peaks were at 966 cm^{-1} and 911 cm^{-1}, respectively. The FTIR spectra of HTPB-co-HEMA were represented by the light orange line in Figure S2a, with an absorption peak at 1725 cm^{-1} that corresponded to the absorption vibration peak of the carbonyl ester of HEMA. The stretching vibration of C(=O)-O-C in the HEMA structural unit generated the peak at 1165cm^{-1}. Stretching vibration peaks of the hydroxyl group linked to HTPB and HEMA were overlapped in the broad absorption band about 3400 cm^{-1}. The stretching vibration peak of the C=C double bond of olefin at 1642 cm^{-1} disappeared, and the absorption peaks at 966 cm^{-1} and 911 cm^{-1} were significantly weakened, indicating that HTPB-co-HEMA was successfully prepared. The two peaks at 1163 $cm^{-1}$and 1073 cm^{-1} in Figure S2a FTIR spectra of the P(HHIP) were identified as the asymmetric stretching vibration peak and the symmetric stretching vibration peak of Si-O-Si, respectively. The weakening absorption peak at 3400 cm^{-1} and the appearance of characteristic absorption peak of secondary amide at 1556 cm^{-1} which displayed the formation of carbamate bonds, proved that IPDI/T_7-POSS has successfully cross-linked with HTPB-co-HEMA. The micro-nanostructures on surface of Ni foam were constructed by P(HHIP) (Figures S2b and S2c).

1.4 The wettability propriety of P(HHIP)@SiO_2@Ni foam

The contact angle and sliding angle of water droplets (oil droplets) of sample were measured at five different positions. The volume of water droplets (oil droplets) was about 5 μL.

Supplementary Section 2: Inhibiting hydrate nucleation test

2.1 The formation of tetrahydrofuran hydrate

As shown in Figure S4a, the experimental device is made up of three sections: a cooling system, hydrate

reaction system and data acquisition system. Data acquisition system: The temperatures of hydrates nucleation were monitored using resistance temperature detectors (RTDs) and resistance thermometers (PT100, ± 0.1 °C). All experimental data were collected using a data acquisition system (DAQ, Model: 34970A, Agilent Technologies) at 10 second intervals.

Cooling system: 60 wt% ethylene glycol aqueous solution was used as refrigerant. Connect the inlet and outlet of the refrigerator with built-in circulation to the outlet and inlet of the jacket beaker in the reaction system, respectively.

Reaction system: The cooling system was in charge of maintaining the reaction temperature in the jacketed beaker. The reaction test tube was inserted in the jacketed beaker's bottom. A magnetic stirrer was placed beneath the jacketed beaker. Magnetic stirring was activated to evenly disseminate the solution and simulate the flow in the pipelines. (The 250 mL jacketed beaker with a diameter of 10 cm and a height of 10 cm)

2.2 Measurement of induction time and evaluation of inhibition performance

Test steps of induction time: (1) The temperature of the cooling system was kept at 0 °C. (2) A 10ml centrifuge tube was filled with 5 g of THF aqueous solution and the cleaned rotor. The sample was then immersed in THF aqueous solution and fixed in a centrifuge tube, with the RTD probe placed beneath the edge of the samples to detect the reaction temperature. (3) After the cooling system temperature was stable, the stirrer was turned on at a speed of 300 r/min. (4) The temperature and time were recorded by the data collecting equipment, which was used to calculated the hydrate nucleation induction time.

THF hydrate could be generated at normal pressure of about 4.4 °C in the molar composition of THF · $17H_2O$. Because hydrate formation is an exothermic process, the induction time can be determined by observing the rapid temperature variations (Figure S5a). The hydrate induction time was the time between when the temperature dropped to the THF hydrate phase equilibrium temperature (defined as t_0) and when the temperature (defined as t_n) began to rise immediately. The induction time (t_i) was $t_n - t_0$, and the subcooling was $\Delta T = T_n - T_0$. Temperature stability was regarded as a sign that the system had stabilized. If no hydrates were generated after 250 min, the induction time was arbitrarily assigned as >250 min. As indicated in Figure 4a, around 30 induction time data points (Figure S5b) were collected to get the average value for each sample. N_t represents the number of samples at time t that had not formed hydrates (assessed by induction time, t_i), and N_0 is the total number of samples. As a result, N_t/N_0 (i.e., the uncrystallized fraction) vs. time represents the inhibition activity of the samples, with the slower the curve declines, the stronger the inhibition performance.[1]

2.3 Online observation of the hydrate nucleation

It was discovered that hydrate clusters first appeared on the surface of Ni foam (Figure S6a(i)), then grew to the bottom of the test tube (Figures S6a(ii-iii)), and then formed hydrates instantly through online observation of the hydrate nucleation process on the surface of the samples (Figures S6a and S6b). While for the P(HHIP)@SiO_2@Ni foam， the THF hydrate expanded from the bottom of the test tube to the gas-liquid interface (Figures 6b(i-ii)), and the crystal clusters quickly increased and surrounded the P(HHIP)@SiO_2@Ni foam surface. The P(HHIP)@SiO_2@Ni foam surface had not entirely nucleated, even if the gas-liquid interface had nucleated.

2.4 Differential Scanning Calorimetry Measurements

It has been reported that DSC is an effective method for investigating the formation and dissociation of hydrates.[2-3]The curves of heat flow could be used to describe the heat (exothermic) released by hydrate formation or the energy (endothermic) required from the system for hydrate decomposition.

DSC test steps: The sample (~10mg) was immersed in 19.1 wt% THF holding in an aluminum sample pan. The sample pan and a reference pan were then placed in the DSC head. It cooled down from 25 °C to - 40°C at a rate of 3 °C/min, held at -40 °C for 3min. and then heated up again to 25 °C at a rate of 3 °C/min under 80 ml/min of nitrogen.

2.5 In situ Raman spectroscopy and Raman Analysis

Raman spectroscopy has been widely applied to investigate hydrate structure, hydration number, and cage occupancy at the molecular level.[4-7] The intensity of a Raman band is proportional to the concentration of the functional group to which it is connected. Samples were immersed in 19.1 wt% THF solution and held in a temperature-controlled stage (Linkam THMS600) for gradient cooling during the Raman test method. Renishaw WiRE 3.4 software was used to record the mode of OH stretching near the surface of samples during the hydrate nucleation or decomposition process. The 532 nm Ar-ion laser was focused as much as feasible on the surface of samples to clarify the effect of samples on induction time. The silicon (Si) crystal standard of 520.7 cm^{-1} was employed to calibrate the subtractive spectrograph. The exposure time was set as 10 s at 10% laser power (∼20 mW) and 10 accumulations (number of scan repetitions per measurement). To analyze the relative strengths of hydrogen bonding in the surface of samples and the THF hydrate structure, *In-situ* Raman spectroscopy with a single Monochromator of 1800 grooves/mm grating and a multichannel air-cooled CCD (charge coupled device) detector was used.

The in-situ Raman test procedures： Firstly, the sample was immersed in 19.1 wt% THF solution, and the temperature of system was gradually reduced from 25 °C to 4.4 °C with cooling at a rate of 5 °C /min, with the temperature remaining at 4.4 °C for 1 min. Cool from 4.4 °C to 0.4 °C at a rate of 1 °C/min, then hold 0.4 °C for 10 min. Keep cooling down from 0.4 °C to -30 °C with cooling at a rate of 1 °C /min, hold -30 °C for 10 min. Steps in the Raman breakdown of THF hydrate: heat up from -30 °C to 25 °C at a rate of 5 °C/min.

Supplementary Section 3: Anti-hydrate-adhesion test

The adhesion force between cyclopentane hydrate particles with different samples were measured by the MMF device. As shown in Figure S4b, the MMF consisted of Zeiss(S100) inverted optical microscope, manual micromanipulator and remote mechanical micromanipulators, a temperature control system (Huber CC1-K20, Germany) and an image processing system. The adhesive force recording device was connected to the camera via the microscope. The cold tank was constructed with aluminum cooling jacket, which was used to control the temperature of the test system.

The MMF measurement process: (1) Cool the temperature of pure cyclopentane to 3.2 °C in a cold tank. (2) Water droplets were placed onto the ends of glass fibers and submerged in liquid nitrogen until frozen, then the ice particle was fastened on the moveable cantilever. Immerse the moveable cantilever into cyclopentane (3.2 °C) holding for 1h to prepare CP hydrate particles. (3) the sample was fixed on the stationary cantilever, then put it into cyclopentane (3.2 °C). (4) Move the moveable cantilever to the sample surface. Hold the samples in contact with the hydrate particles for 10s with 0.1 μN preload force. (5) following the contact period, hydrates particle was pulled-off from the sample surface at a constant velocity until it detached from the sample surface. Based on 40 repeated tests,

the average value presented in this work.

Figure S8 shown the displacement (Δx) of CP hydrate with different samples under the same experimental circumstances. Stainless steel had the greatest displacement, followed by Ni Foam, P(HHIP)@SiO_2@SS. P(HHIP)@SiO_2@Ni foam was the smallest with displacement (Δx) near to zero. Each sample was measured in 40 groups at different positions, with an average value calculated. According to Hook's Law Eq. (1), the adhesive force (F) between materials and hydrate particles is proportional to adhesive displacement (x) and spring constant of the fiberglass (k).

$$F=k*x \tag{1}$$

$$F=\frac{3E\pi D^4}{64L^3} \tag{2}$$

$$F_a=F/R \tag{3}$$

The spring constant of the fiberglass can be calculated according to Eq. (2), where E is the elastic modulus of fiberglass, D is the diameter of fiberglass, and L is the length of fiberglass, the value of E is 70 GPa in this experiment.[8] The standardized adhesion force F_a was used instead of F for eliminating the influence of particle size on adhesive forces, as Eq. (3), where R is the radius of hydrate particles.

Supplementary Section 4: Mechanical and chemical stability tests

1. Erosion resistance test

Erosion resistance test was conducted in a 2.5 L beaker containing 180 g sands and 1 L water. Samples were submerged in the sediment fluid, which was stirred with speed of 1500 rpm to accelerate scouring. Water droplets rolled away from the surface of materials before abrasion (Fig. S9a, 0h). P(HHIP)@SiO_2@SS had lost super-hydrophobicity after eroded for 0.5 h. Teflon tape had worn away (Fig. S9a, 3h), but P(HHIP)@SiO_2@Ni foam still possessed the super-hydrophobicity after be eroded for 4h (Fig. S9a, 4h, Supplementary Video 1).

2. Scratch test

To prove the erosion resistance of the P(HHIP)@SiO_2@Ni foam, the scratch test (knife scraping) was conducted, as shown in Supplementary Video 2. The surface of P(HHIP)@SiO_2@Ni foam (110ppi with a size of 1 cm × 2.5 cm × 0.13 cm) was scraped at least 80 times with a knife. After the test, the water drops could roll off the abrasion surface without residues, no water drops pinned into its surface. The wettability of the abrased samples was characterized as shown in Figure S10a.

3. Friction test

To further prove the mechanical robustness of the P(HHIP)@SiO_2@Ni foam (110ppi with a size of 1 cm × 2.5 cm × 0.13 cm), the load sliding reciprocating friction testing was carried out, as shown in Supplementary Video 3. A loading weight (50 g) on the surface of the P(HHIP)@SiO_2@Ni foam was ragged forward and backward as one cycle, repeatedly at least 60 times. The wettability of the abrased samples was characterized as shown in Figure S10b.

4. The chemical stability test

To study the chemical stability of samples when exposed to low and high pH values, the samples were placed in sodium hydroxide solution (pH=10, pH=14), hydrochloric acid solution (pH=1, pH=4) and water (pH=7). The variation of the contact angle of the samples surface with immersion time was recorded.

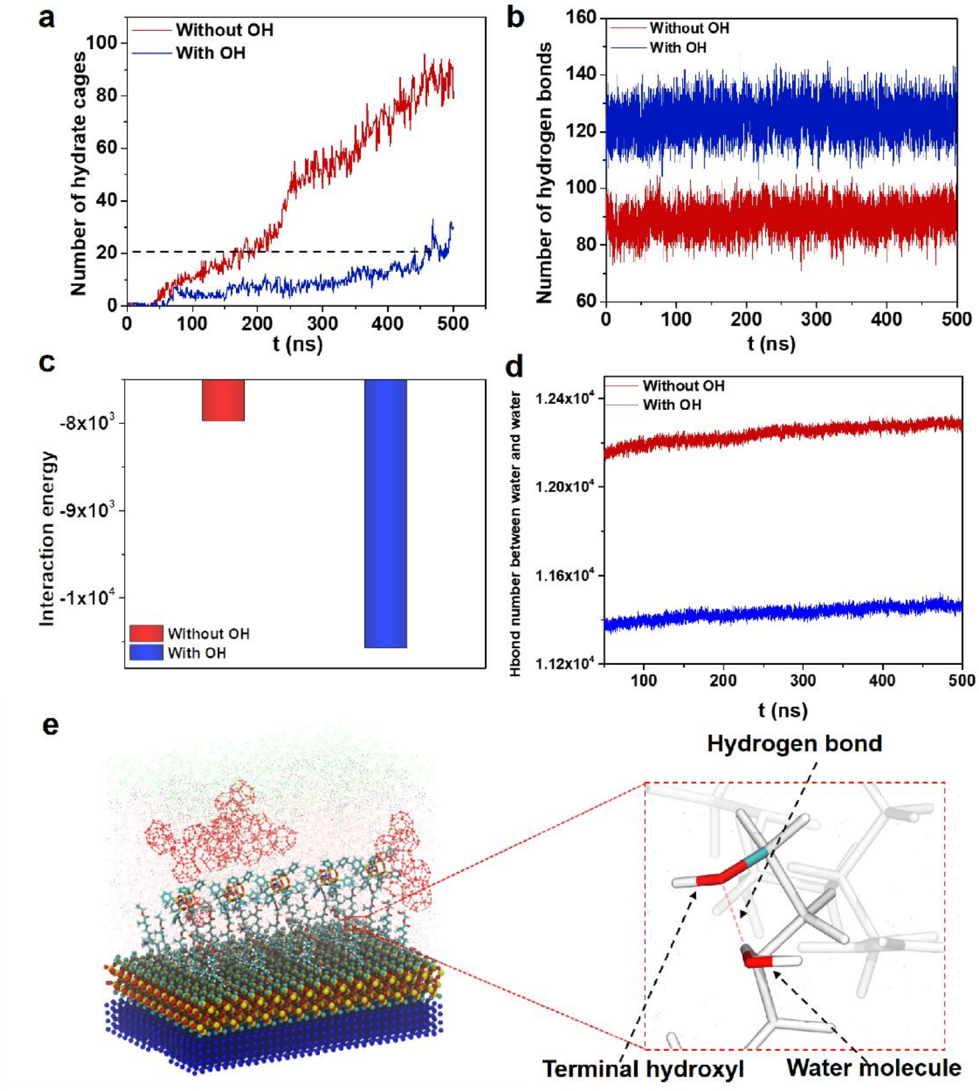

Figure S1. Molecular Dynamics Simulations predicting the effects of terminal hydroxyl groups on hydrate formation at 250 K and 500 bar. a) Comparison of the number of hydrate cage showing the effect of hydroxyl groups on hydrate formation. b) Calculated the interaction energy between water molecular and samples surface to character effect of functional groups of samples surface on hydrate formation. c) Measured the number of hydrogen bond between water molecular and samples surface. d) Comparison of the number of hydrogen bond between water molecular and water molecular showing the effect of hydroxyl groups on the formation of hydrogen bond during hydrate formation. e) Illustration of hydrogen bond formed between water molecule and hydroxyl groups.

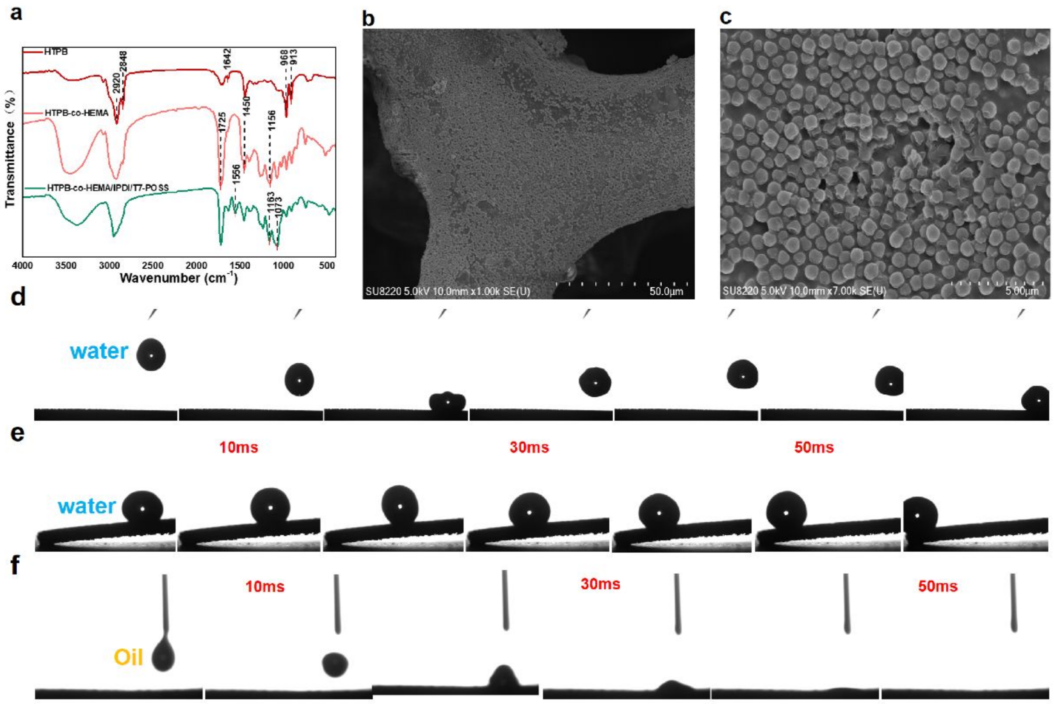

Figure S2. The characteristics of the P(HHIP)@SiO_2@Ni foam. a) The FTIR spectra of HTPB (the dark red line), HTPB-co-HEMA (the light orange line) and P(HHIP) (light blue line). b-c) The Scanning electron micrographs showing micro-scaled skeleton and the micro-nanostructure topography of P(HHIP) @Ni foam. Low-magnification SEM image (b) Showing surface morphology of micro-scaled skeleton. High-magnification SEM image (c) Showing the micro-nanostructure topography constructed by T_7-POSS. d) Snapshots of water droplet dropped from a height of 1 cm and rebounded from P(HHIP)@SiO_2@Ni foam. e) The snapshots of water droplet slid from the surface of P(HHIP)@SiO_2@Ni foam (sliding angle 5 ± 2°). f) Snapshots of oil droplets quickly wetted the surface of P(HHIP)@SiO_2@Ni foam within 0.05 seconds.

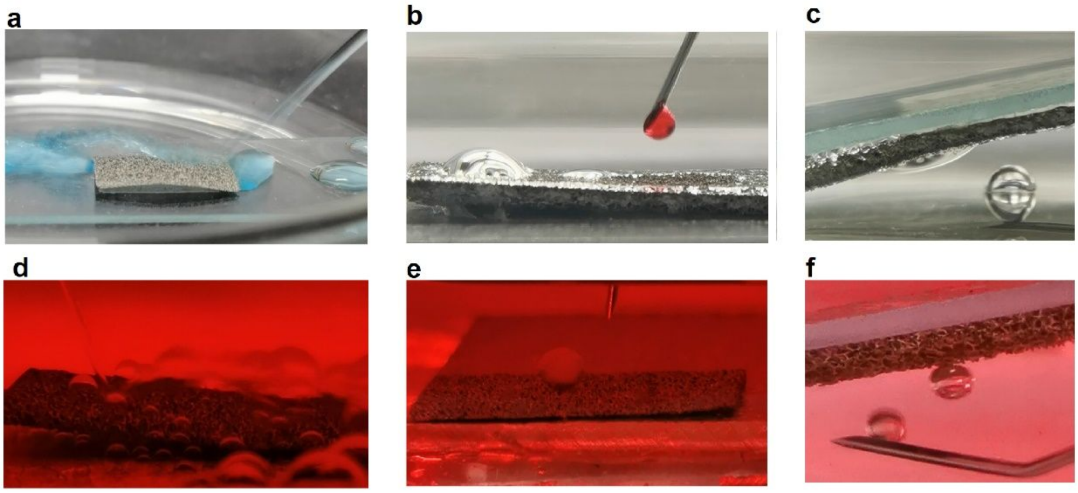

Figure S3. The surface wetting of the P(HHIP)@SiO_2@Ni foam. a) The water jet bounced off in the air. b) The super-lipophilicity of P(HHIP)@SiO_2@Ni foam under the water. c) The super-aerophilicity of P(HHIP)@SiO_2@Ni foam under the water. d) The water jet bounced off in the cyclopentane solution. e) The superhydrophobic of P(HHIP)@SiO_2@Ni foam in the oil. f) The super-aerophobicity of P(HHIP)@SiO_2@Ni foam in the oil.

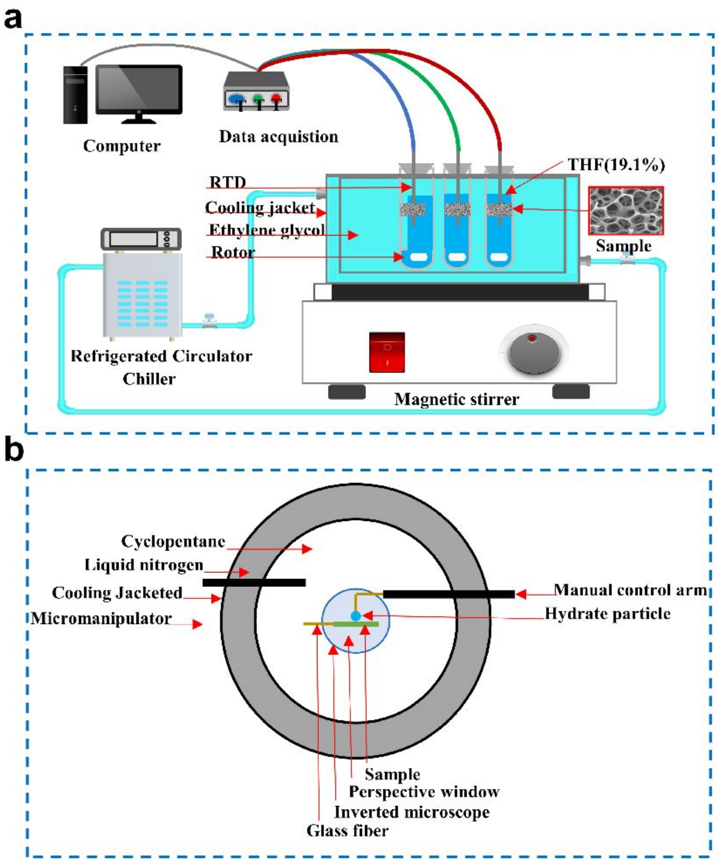

Figure S4. The schematic diagram of experimental apparatus in this study. a) Schematic diagram of hydrate induction time test device. b) Schematic diagram of hydrate adhesion force test device.

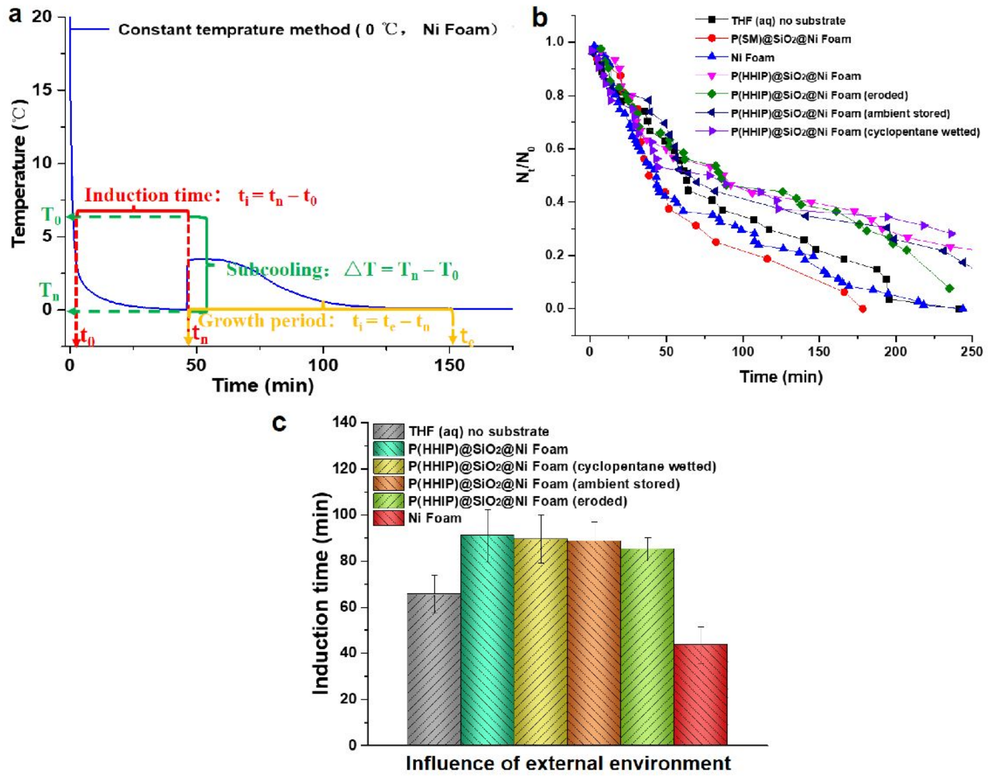

Figure S5. The measuring of induction time. a) The typical curve for measuring induction time for THF hydrate formation. b) Comparison of induction effects of different external environment on the formation of THF hydrate at 273.2 K. c) Comparison of mean induction time of the P(HHIP)@SiO_2@Ni foam in different external environment.

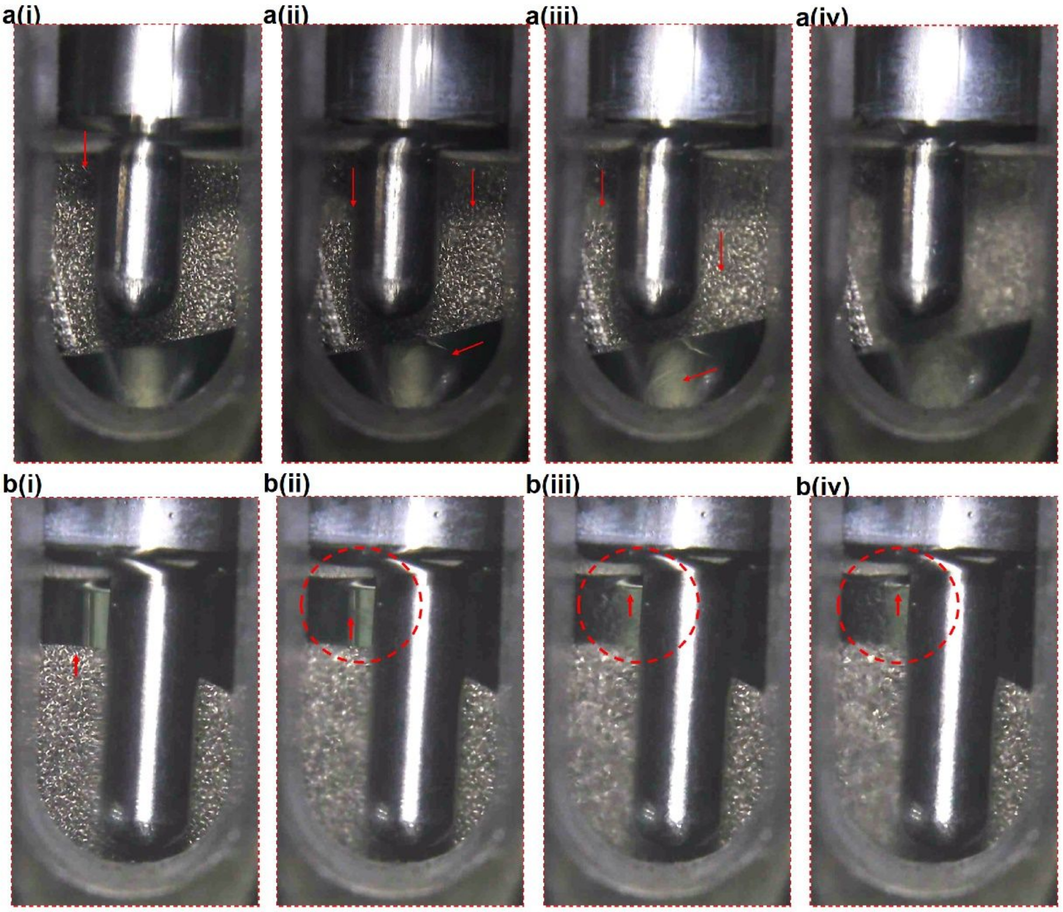

Figure S6. Slow motion screenshot at the moment of hydrate nucleation. a) Morphology changes of on uncoated Ni foam surface during the THF hydrate formation. b) Morphology changes of the P(HHIP)@SiO_2@Ni foam during the THF hydrate formation. (Under the atmospheric and 273.2 K. The direction of the red arrow was the growth direction of the THF hydrate.)

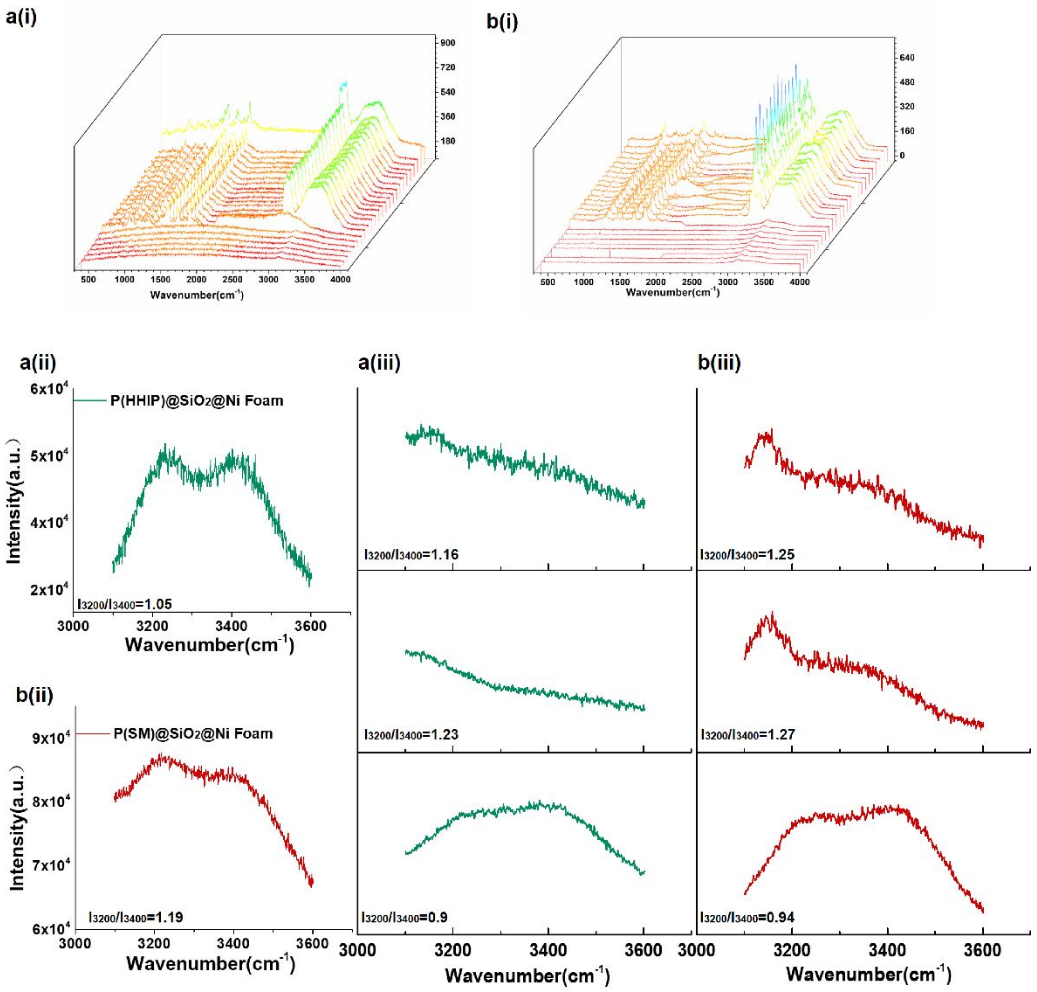

Figure S7. The Raman spectra of different samples surface. a) P(HHIP)@SiO_2@Ni Foam, b) P(SM)@SiO_2@Ni Foam. (i) During hydrate formation, the Raman spectra of different materials surface. (ii) Raman spectra and $R_{OH\text{-}W}$ of different materials in water (25 °C). (iii) The Raman spectra and $R_{OH\text{-}H}$ of different material surface in a 19 wt % THF aqueous solution during nucleation process.

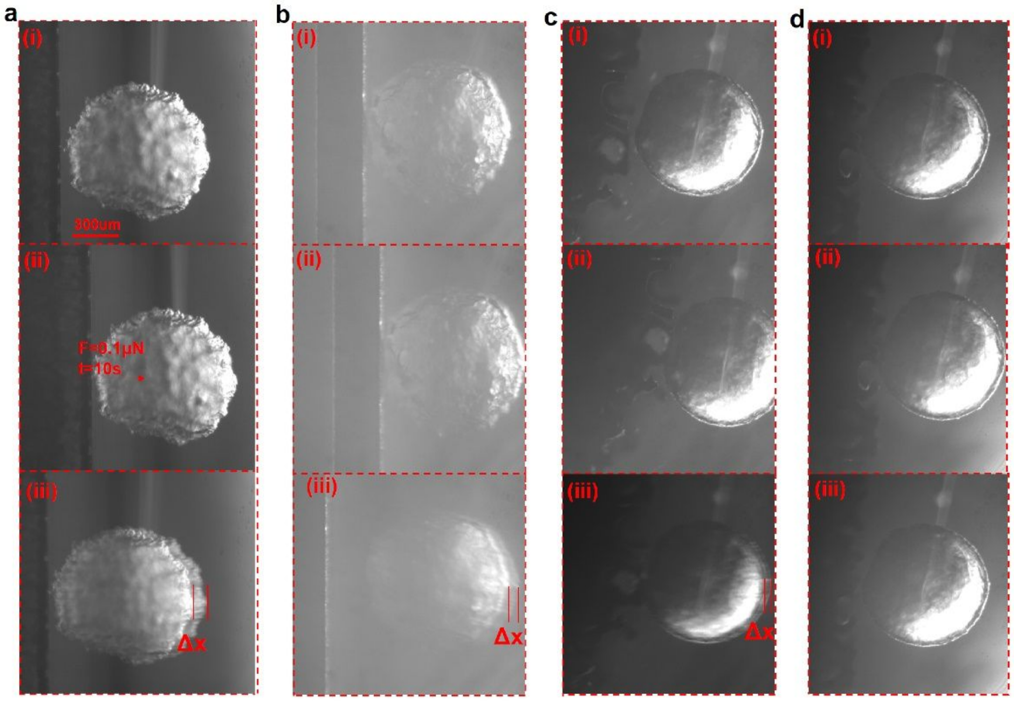

Figure S8. The Example diagram of adhesion test process for different samples. a) SS. b) P(HHIP)@SiO_2@SS. c) Ni foam. d) P(HHIP)@SiO_2@Ni foam. (i) Before the material was moved to the CP hydrate particle. (ii) The material was giving a preload force (0.1 μN) with a contact time of 10s. (iii) The material and the CP hydrate particle was separated, the displacement (Δx) was obtained. Usually, under the same experimental conditions, the longer displacement (Δx), which is denoted as the migration distance of CP hydrate particles from the moment of contact to that of separation, the stronger the adhesion force will be.

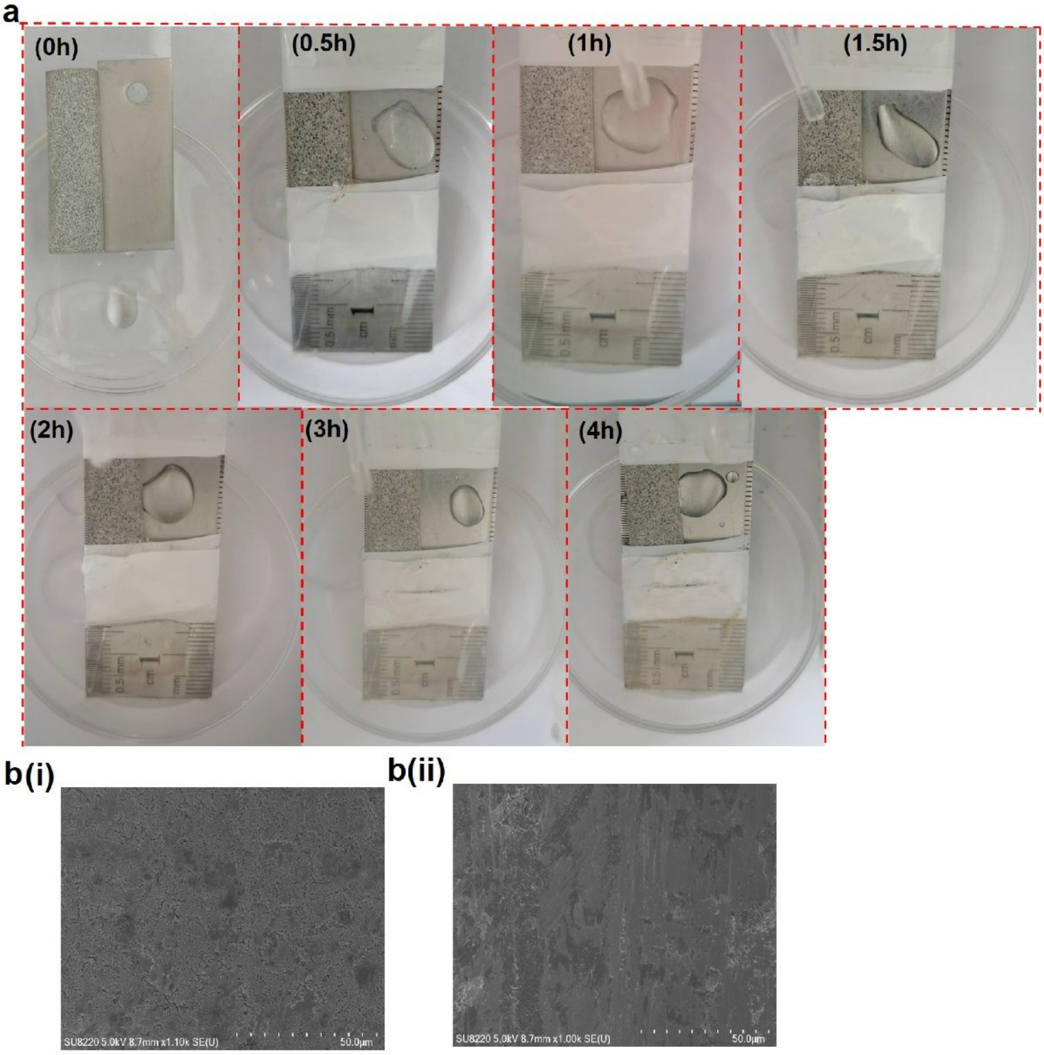

Figure S9. The anti-erosion of different materials. a) The change of different wettability samples surfaces versus scouring time. b) The SEM of P(HHIP)@SiO_2@SS eroded (i) Before the P(HHIP)@SiO_2@SS was eroded. (ii) After the P(HHIP)@SiO_2@SS was eroded 30 mins with stirring speed of 1500 rpm.

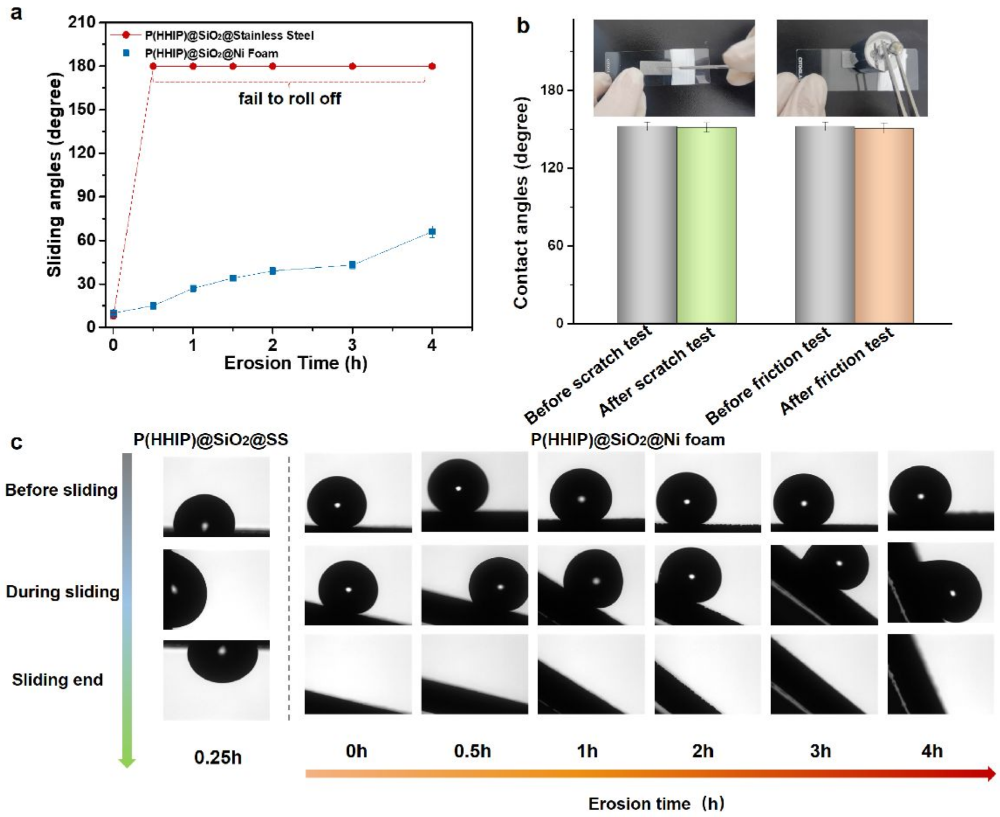

Figure S10. The anti-erosion performance of materials. a) The variation of the sliding angle of different samples surface with the erosion time. b) To prove the erosion resistance of the P(HHIP)@SiO_2@Ni foam, the scratch test and the load sliding reciprocating friction testing were carried out. c) The diagram of the sliding process of different samples surface with the erosion time.

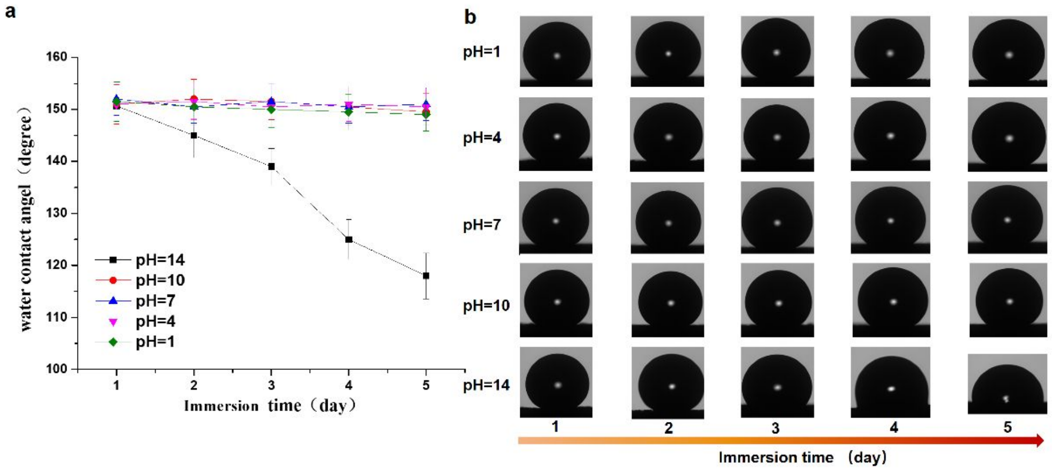

Figure S11. The chemical stability of materials. a) The wettability of the surfaces exposed to solutions with low and high pH values. b) The diagram of the contact angles of samples surfaces in different pH values solutions with the immersion time.

Table S1

The anti-hydrates-adhesion performance of superhydrophobic materials in CyC5 hydrate system.

Substrate	Coating	Contact angle/°	Adhesion force	Decrease percentage/%	Refs.
Carbon steel	Wax	170.7 ± 3.1	18.4 ± 2.9 kPa	93.7	[9]
Carbon steel	PTFE	164.0 ± 3.1	13.1 ± 1.75 kPa	95.5	[10]
Steel	pPFDA/pDVB	157.0± 4.5	34 ± 12 kPa	84.5	[11]
X90	CuO	160 ± 3.1	0.13 mN	>91.9	[12]
X80	CeO_2/pDA	154.7± 0.8	0.001 mN/m	98.9	[8]
Steel	Graphite	154±7	0.85 mN/m	79	[13]
Cu	Graphite fluoride	150	0.01 mN/m	>99.7	[14]
3D porous Ni foam	P(HHIP)@SiO_2	151±3	0.006 mN/m	98.7	This work

Supplementary Videos (1-3)

Supplementary Video 1：Anti-erosion test.

Supplementary Video 2：Anti-scratch test.

Supplementary Video 3：Anti-abrasion test.

References

1. Zeng, H.; Wilson, L. D.; Walker, V. K.; Ripmeester, J. A., Effect of Antifreeze Proteins on the Nucleation, Growth, and the Memory Effect during Tetrahydrofuran Clathrate Hydrate Formation. *J. Am. Chem. Soc.* **2006,** *128* (9), 2844-2850.
2. Lachance, J. W.; Dendy Sloan, E.; Koh, C. A., Effect of hydrate formation/dissociation on emulsion stability using DSC and visual techniques. *Chem. Eng. Sc.* **2008,** *63,* (15), 3942-3947.
3. Maeda, N.; Kelland, M. A.; Wood, C. D., Ranking of kinetic hydrate inhibitors using a high pressure differential scanning calorimeter. *Chem. Eng. Sci.* **2018,** *183,* 30-36.
4. Braeuer, A.; Hankel, R. F.; Mehnert, M. K.; Schuster, J. J.; Will, S., A Raman technique applicable for the analysis of the working principle of promoters and inhibitors of gas hydrate formation. *J. Raman Spectrosc.* **2015,** *46,* (11), 1145-1149.
5. Zeng, X.-Y.; Zhong, J.-R.; Sun, Y.-F.; Li, S.-L.; Chen, G.-J.; Sun, C.-Y., Investigating the partial structure of the hydrate film formed at the gas/water interface by Raman spectra. *Chem. Eng. Sci.* **2017,** *160,* 183-190.
6. Kumar, A.; Veluswamy, H. P.; Linga, P.; Kumar, R., Molecular level investigations and stability analysis of mixed methane-tetrahydrofuran hydrates: Implications to energy storage. *Fuel* **2019,** *236,* 1505-1511.
7. Li, H.; Stanwix, P.; Aman, Z.; Johns, M.; May, E.; Wang, L., Raman Spectroscopic Studies of Clathrate Hydrate Formation in the Presence of Hydrophobized Particles. *J. Phys. Chem. A* **2016,** *120* (3), 417-24.
8. Zhang, W.; Fan, S.; Wang, Y.; Lang, X.; Li, G., Preparation and performance of biomimetic superhydrophobic coating on X80 pipeline steel for inhibition of hydrate adhesion. *Chem. Eng. J.* **2021,** *419,* 129651.
9. Liu C.; Zeng X.; Yan C.;Zhou C.; Li M.;Wang Z.; Effects of solid precipitation

and surface corrosion on the adhesion strengths of sintered hydrate deposits on pipe walls. *Langmuir,* **2020***, 36* (50), 15343-15351.

10. Liu C.; Wang Z.; Tian J.; Yan C.; Li M., Fundamental investigation of the adhesion strength between cyclopentane hydrate deposition and solid surface materials. *Chem. Eng. Sci.* **2020,** *217.* 115524

11. Sojoudi, H.; Arabnejad, H.; Raiyan, A.; Shirazi, S. A.; McKinley, G. H.; Gleason, K. K., Scalable and durable polymeric icephobic and hydrate-phobic coatings. *Soft Matter* **2018,** *14* (18)*,* 3443-3454.

12. Dong, S.; Li, M.; Liu, C.; Zhang, J.; Chen, G., Bio-inspired Superhydrophobic Coating with Low Hydrate Adhesion for Hydrate Mitigation. *J. Bionic. Eng.* **2020,** *17* (5)*,* 1019-1028.

13. Aman, Z. M.; Sloan, E. D.; Sum, A. K.; Koh, C. A., Adhesion force interactions between cyclopentane hydrate and physically and chemically modified surfaces. *Phys. Chem. Chem. Phys.* **2014***, 16* (45)*,* 25121- 25128.

14. Zhang W.; Fan S.; Wang Y.; Lang X.; Li G.; Development of a composite structured surface for durable anti-hydrate and enhancing thermal conductivity. *Int. J. Heat Mass Tran.,* **2022***, 192*, 122909.
